# Transient Receptor Potential Channels TRPM4 and TRPC3 Critically Contribute to Respiratory Motor Pattern Formation but not Rhythmogenesis in Rodent Brainstem Circuits

**DOI:** 10.1523/ENEURO.0332-17.2018

**Published:** 2018-02-09

**Authors:** Hidehiko Koizumi, Tibin T. John, Justine X. Chia, Mohammad F. Tariq, Ryan S. Phillips, Bryan Mosher, Yonghua Chen, Ryan Thompson, Ruli Zhang, Naohiro Koshiya, Jeffrey C. Smith

**Affiliations:** 1 Cellular and Systems Neurobiology Section, National Institute of Neurological Disorders and Stroke, National Institutes of Health, Bethesda, MD 20892; 2 Department of Physics, University of New Hampshire, Durham, NH 03824

**Keywords:** Breathing, dynamic calcium imaging, *I*_CAN_, pre-Bötzinger complex

## Abstract

Transient receptor potential channel, TRPM4, the putative molecular substrate for Ca^2+^-activated nonselective cation current (*I*_CAN_), is hypothesized to generate bursting activity of pre-Bötzinger complex (pre-BötC) inspiratory neurons and critically contribute to respiratory rhythmogenesis. Another TRP channel, TRPC3, which mediates Na^+^/Ca^2+^ fluxes, may be involved in regulating Ca^2+^-related signaling, including affecting TRPM4/*I*_CAN_ in respiratory pre-BötC neurons. However, TRPM4 and TRPC3 expression in pre-BötC inspiratory neurons and functional roles of these channels remain to be determined. By single-cell multiplex RT-PCR, we show mRNA expression for these channels in pre-BötC inspiratory neurons in rhythmically active medullary *in vitro* slices from neonatal rats and mice. Functional contributions were analyzed with pharmacological inhibitors of TRPM4 or TRPC3 *in vitro* as well as in mature rodent arterially perfused *in situ* brainstem–spinal cord preparations. Perturbations of respiratory circuit activity were also compared with those by a blocker of *I*_CAN_. Pharmacologically attenuating endogenous activation of TRPM4, TRPC3, or *I*_CAN_ *in vitro* similarly reduced the amplitude of inspiratory motoneuronal activity without significant perturbations of inspiratory frequency or variability of the rhythm. Amplitude perturbations were correlated with reduced inspiratory glutamatergic pre-BötC neuronal activity, monitored by multicellular dynamic calcium imaging *in vitro.* In more intact circuits *in situ*, the reduction of pre-BötC and motoneuronal inspiratory activity amplitude was accompanied by reduced post-inspiratory motoneuronal activity, without disruption of rhythm generation. We conclude that endogenously activated TRPM4, which likely mediates *I*_CAN_, and TRPC3 channels in pre-BötC inspiratory neurons play fundamental roles in respiratory pattern formation but are not critically involved in respiratory rhythm generation.

## Significance Statement

Biophysical mechanisms generating the timing and patterning of rhythmic respiratory movements in mammals remain largely undefined. Calcium signaling–based theories for respiratory rhythm generation incorporating calcium-activated nonselective cation currents (*I*_CAN_), postulated to be a type of transient receptor potential channel TRPM4, have been proposed but remain unproven. Here, we revealed that TRPM4 and TRPC3 channels are present and functionally active in rodent brainstem respiratory neurons, including in the inspiratory rhythm-generating circuits of the pre-Bötzinger complex. However, we established that these channels are not fundamentally involved in rhythm generation, but critically contribute to the formation of respiratory motor patterns. These results help resolve the longstanding debate in the field about the contributions of *I*_CAN_ to rhythm and pattern generation in respiratory circuits.

## Introduction

Members of the transient receptor potential (TRP) channel superfamily, which mediate cationic current fluxes and control cell excitability and intracellular signaling, are involved in diverse aspects of brain function. Here we investigated roles of two subtypes of TRP channels—TRPM4 of the melastatin TRPM channel family and TRPC3 of the canonical TRPC channel family—in generating respiratory motor activity in the rodent brainstem–spinal cord. We established that these channels are expressed at the molecular level in populations of respiratory interneurons and motoneurons, they are endogenously activated during circuit activity, and they have a fundamental role in respiratory motor pattern generation.

Prominent, but unproven, Ca^2+^-based theories involving TRPM4 have been proposed for respiratory rhythm generation (Mironov, 2009; Del Negro et al., 2010) by excitatory neurons in the mammalian brainstem pre-Bötzinger complex (pre-BötC), the established locus of interneurons in the medulla critical for inspiratory rhythm generation (Smith et al., 1991; Feldman and Del Negro, 2006). TRPM4, known to be a Ca^2+^-activated TRP channel (Guinamard et al., 2014), is postulated to be the molecular substrate of Ca^2+^-activated nonselective cation current (*I*_CAN_) in respiratory neurons (Crowder et al., 2007; Del Negro et al., 2010). TRPM4-mediated *I*_CAN_ is proposed to be importantly involved in rhythm generation by functionally coupling activity-dependent intracellular Ca^2+^ signaling to neuronal depolarization and rhythmic neuronal activity generation (Del Negro et al., 2010; Guinamard et al., 2010). Previous studies (e.g., Koizumi and Smith, 2008) have shown that the rhythmically active neurons in pre-BötC circuits as well as neurons in downstream rhythmic drive transmission circuits exhibit large transient increases of intracellular Ca^2+^, potentially mediating TRPM4/*I*_CAN_ channel activity, during each respiratory cycle. TRPC3, on the other hand, is not directly activated by Ca^2+^, but mediates Na^+^/Ca^2+^ fluxes, and as a proposed Ca^2+^ store-operated channel, may be involved in regulating neuronal Ca^2+^-related signaling (Talavera et al., 2008; Birnbaumer, 2009; Guinamard et al., 2013), potentially affecting TRPM4/*I*_CAN_ in respiratory neurons. The role of TRPC3-mediated cationic currents/Ca^2+^-related signaling in generating respiratory neuron activity has not been investigated.

TRPM4 and TRPC3 channels have been identified by mRNA/protein expression in tissue harvested from the mouse pre-BötC region (Crowder et al., 2007; Ben-Mabrouk and Tryba, 2010), although they have not been demonstrated to be expressed in functionally identified respiratory neurons. Here we examined, by single-cell RT-PCR, expression of TRPM4 and TRPC3 mRNA in functionally identified glutamatergic, glycinergic, and GABAergic inspiratory pre-BötC neurons as well as inspiratory motoneurons. We also examined neuronal channel expression by antibody labeling in the pre-BötC region and motor nuclei. We then investigated whether these channels contribute to respiratory circuit activity by pharmacological experiments with the selective channel inhibitors 9-phenanthrol for TRPM4 (Guinamard et al., 2014) and 3-pyrazole for TRPC3 (Kiyonaka et al., 2009), in both neonatal rat and mouse slice preparations with rhythmically active pre-BötC and respiratory motor circuits *in vitro.* Comparative analyses for these two species was important because there are numerous studies on respiratory rhythm and pattern generation using rats (e.g., Gray et al., 2001; Onimaru and Homma, 2003; Koizumi and Smith, 2008) or mice (e.g., Thoby-Brisson and Ramirez, 2001; Peña et al., 2004; Del Negro et al., 2005), so it is necessary to establish commonality of Ca^2+^-based mechanisms for respiratory rhythm and pattern generation. We also analyzed perturbations of pre-BötC excitatory circuit activity by dynamic Ca^2+^ imaging of inspiratory glutamatergic pre-BötC neurons with a genetically encoded Ca^2+^ sensor (Chen et al., 2013) in transgenic mice. We show that amplitudes of inspiratory pre-BötC neuronal activity, and the correlated amplitudes of motoneuronal output *in vitro,* are significantly reduced by TRPM4 and TRPC3 channel inhibitors. The pharmacological profile of inspiratory activity attenuation by inhibiting TRPM4 activation matched that with another proposed blocker of *I*_CAN_, flufenamic acid (FFA), consistent with the concept that TRPM4 mediates *I*_CAN_ (Launay et al., 2002). In all cases, the attenuation of inspiratory circuit activity occurred without significant perturbations of the frequency of the inspiratory rhythm.

We also demonstrate critical involvement of TRPM4 and TRPC3 channels in regulating the amplitude of pre-BötC population activity and motor output patterns in more intact respiratory circuits active in arterially perfused brainstem–spinal cord *in situ* preparations from mature rats and mice. The reduction, by the channel inhibitors, of pre-BötC and motoneuronal inspiratory activity amplitude recorded electrophysiologically was accompanied by reductions of post-inspiratory motoneuronal activity. These amplitude perturbations also occurred without disrupting rhythm generation. In general, our results indicate that endogenous activation of these two types of TRP channels are involved in generating respiratory motor patterns, but critically not rhythm generation, in both neonatal and mature rodents.

## Materials and Methods

### Animal procedures

All animal procedures were approved by the Animal Care and Use Committee of the National Institute of Neurological Disorders and Stroke.

### Immunohistochemical labeling of TRPM4 and TRPC3 channels

We examined fluorescence antibody labeling for TRPM4 and TRPC3 channels to identify channel expression in pre-BötC neurons in neonatal and mature rats and mice. In addition, we examined channel expression in relation to specific neurotransmitter phenotypes of neurons within the pre-BötC, BötC, and rostral ventral respiratory group (rVRG) regions. We used transgenic Cre-driver mouse strains crossed with Cre-dependent reporter transgenic strains to express fluorescent protein (tdTomato) in excitatory or inhibitory neurons by the cell type–specific promoters (Gong et al., 2007) vesicular glutamate transporter type-2 (VgluT2) or glycine transporter type-2 (GlyT2): VgluT2-tdTomato for glutamatergic neurons, and GlyT2-tdTomato for glycinergic neurons. The VgluT2-tdTomato strain was produced by crossing a VgluT2-ires-Cre strain (Slc17a6^tm2(cre)Lowl^/J, IMSR JAX: 016963, RRID: IMSR_JAX: 016963, Jackson Laboratory) with a Cre-dependent tdTomato reporter strain [B6.Cg-Gt(ROSA)26Sor^tm9(CAG-tdTomato)Hze^/J, also called Ai9(RCL-tdT), IMSR JAX: 007909, RRID: IMSR_JAX: 007909, Jackson Laboratory]. The GlyT2-tdTomato mouse line was produced by crossing a GlyT2-Cre line [B6.FVB(cg)-Tg(Slc6a5-cre)KF109Gsat/Mmucd, MMRRC 036055-UCD, RRID: MMRRC_036055-UCD, MMRRC, University of California, Davis] with the Ai9(RCL-tdT) line. In each of these double transgenic lines, we analyzed colabeling by TRPM4 or TRPC3 channel antibody in neurons prelabeled with tdTomato to identify expression of each channel in glutamatergic or glycinergic neurons.

The medulla oblongata from neonatal and mature rats or mice was fixed in 4% paraformaldehyde (wt/vol) in PBS, cryoprotected overnight at 4°C in 30% sucrose and 0.1 m PBS solution, and sectioned coronally (25 or 50 µm) with a freezing microtome. For fluorescent immunohistochemistry, floating sections were incubated with 10% donkey serum in PBS with Triton X-100 (0.3%) and incubated for 48–72 h at room temperature with the following primary antibodies: polyclonal rabbit anti-TRPM4 (ab63080, Abcam ab63080, RRID: AB_956418, 1:1000) and polyclonal rabbit anti-TRPC3 (ACC-016, Alomone Labs, ACC-016, RRID: AB_2040236, 1:200). We verified the specificity of these TRPM4 and TRPC3 antibodies by confirming the absence of immunoreactivity in the mouse medullary tissue sections with the primary antibody that was preincubated for 1 h at room temperature with saturating concentrations (10:1) of the antigenic blocking peptide (TRPM4: ab65597, Abcam, TRPC3: ACC-016, Alomone Labs). We also note that the specificity of the same TRPM4 and TRPC3 antibodies as those we used has been confirmed in a TRPM4 knockout mouse (Schattling et al., 2012) and a TRPC3 knockout mouse (Feng et al., 2013), respectively. Individual sections were then rinsed with PBS and incubated for 2 h with the secondary antibody (donkey anti-rabbit, Dylight 647). Individual sections were mounted on slides and covered with an anti-fading medium (Fluoro-Gel; Electron Microscopy Sciences). Fluorescent labeling of neurons was visualized with a laser-scanning confocal imaging system (Zeiss LSM 510). Motoneurons were identified by antibody labeling for choline acetyltransferase (ChAT; goat anti-ChAT, Millipore AB144, RRID: AB_90650, 1:200; donkey anti-goat-Dylight 488, 1:500). TRP channel expression in cell bodies of interneurons was identified by the presence of channel immunoreactivity without ChAT antibody labeling. All images were color/contrast enhanced and adjusted with a thresholding filter in Adobe Photoshop.

For tallying the numbers of TRPM4 or TRPC3 channel antibody-labeled neurons in adult (3–5-mo-old) transgenic mice with glutamatergic or glycinergic neurons labeled with tdTomato fluorescent protein as presented in Results, we counted labeled neurons within a region (300–400 µm diameter depending on animal size, ventral to the nucleus ambiguus) in the coronal plane of 25-µm-thick tissue sections obtained from the pre-BötC, BötC, or rVRG regions on both sides of the medulla. The locations and rostro-caudal extent of each region were defined from our established anatomic criteria based on previous electrophysiological recording/cell activity mapping studies (e.g., Koizumi et al., 2016) as well as the present neuronal population activity recordings in the adult transgenic mouse *in situ* brainstem–spinal cord preparations. The anatomic criteria included the location, in the ventrolateral medullary reticular formation, of the BötC region at the levels of the compact division of nucleus ambiguus (NAc), the pre-BötC region at the levels of the semicompact division of NA (NAsc), and the rVRG region extending from near the level of obex to the caudal end of the pre-BötC. The rostro-caudal dimension of the pre-BötC region was ∼350 μm; the BötC region, ∼550 µm; and the rVRG region, ∼500 µm in the 3–5-mo-old adult mice used for the analysis. We selected 4–6 of the coronal sections from each region so that sections at different levels clearly within the region were included for the bilateral cell counting, and our sample included sections from both the caudal and rostral half of each region to produce the regional tally of labeled neurons presented.

### Rhythmically active medullary slice preparations *in vitro*

Rhythmically active transverse slices of the medulla oblongata that contained the pre-BötC and rostral end of the hypoglossal (XII) motor nucleus with intact XII nerve rootlets (Smith et al., 1991; Koizumi et al., 2013) were cut from neonatal [postnatal day 1–5 (P1–P5)] Sprague-Dawley rats of either sex (350–400-µm-thick slices) or from neonatal (P3–P8) mice of either sex (300–400-µm-thick slices). The slice was superfused (4 ml/min) *in vitro* in a recording chamber (0.2 ml) mounted on the stage of an upright laser scanning microscope with artificial cerebrospinal fluid (ACSF) containing the following (in mm): 124 NaCl, 25 NaHCO_3_, 3 KCl, 1.5 CaCl_2_, 1.0 MgSO_4_, 0.5 NaH_2_PO_4_, 30 d-glucose equilibrated with 95% O_2_ and 5% CO_2_ (pH 7.35–7.40 at 27°C). During experiments, rhythmic respiratory network activity, monitored by recording inspiratory discharge in XII nerve rootlets (see below), was maintained by elevating the superfusate K^+^ concentration to 8–9 mm.

### Calcium imaging and identification of pre-BötC respiratory neurons *in vitro*

We employed Ca^2+^-sensors, either Ca^2+^-sensitive synthetic dye in slices from wild-type (WT) neonatal rats and mice or genetically encoded protein Ca^2+^ sensor with fast kinetics (GCaMP6f; Chen et al., 2013) in transgenic mice, to dynamically image Ca^2+^ activity of pre-BötC neurons for functional identification of respiratory neurons *in vitro*. In experiments with WT neonatal rats or mice, the Ca^2+^ imaging was combined with whole-cell patch-clamp recording from the identified rhythmically active inspiratory neurons. In some of these experiments, the neurons were retrogradely labeled through their contralaterally projecting axons using membrane semipermeable acetoxymethyl (AM) dye (Oregon Green BAPTA-1 am: OGB, Invitrogen) microinjected in the midline region of the slice (Koshiya and Smith, 1999; Koizumi et al., 2013). The slice was incubated overnight (12 h) in ACSF containing antibiotics (500 units/l penicillin, 0.5 mg/l streptomycin, and 1 mg/l neomycin) to allow labeling of pre-BötC neurons and used for optical and electrophysiological recordings throughout the next day. In other experiments, we microinjected the dyes directly into the pre-BötC to label cells nonselectively regardless of their axonal projections (Koizumi et al., 2013). The microinjection pipette (tip size: 2–3 µm) was placed at a depth of 150 µm in the slice and ∼100 µm away from the center of the pre-BötC to avoid excessive dye deposits and high background fluorescence in the imaging area of interest, and OGB was pressure injected (∼20 psi, 2 min). The slice was incubated (>1 h) for sufficient dye loading before the recording experiments.

In the set of imaging experiments with GCaMP6f in transgenic mice, we selectively expressed this Ca^2+^ sensor in glutamatergic neurons using Cre-driven expression controlled by the VgluT2 promoter (VgluT2-GCaMP6f mice). These mice were produced by crossing the VgluT2-iris-Cre strain and a Cre-dependent GCaMP6f expressing strain [B6;129S-*Gt(ROSA)26Sor^tm95.1(CAG-GCaMP6f)Hze^*/J, IMSR JAX: 024105, RRID: IMSR_JAX: 024105, Jackson Laboratory]. We imaged and quantified inspiratory-related GCaMP6f fluorescence transients in the pre-BötC glutamatergic neuronal population, which provides voltage-dependent control of inspiratory frequency and functions as the critical rhythmogenic population (Koizumi et al., 2016).

In all experiments, optical imaging was performed with a Leica multiphoton laser scanning upright microscope (TCS SP5 II MP with DM6000 CFS system, LAS AF software), 20× water-immersion objective (N.A. 1.0), beam-splitter (560 nm), and emission filter (525/50, Semrock). A two-photon Ti:sapphire pulsed laser (MaiTai, Spectra Physics) was used at 800–880 nm for Ca^2+^-sensitive dye or 910–920 nm for GCaMP6f, with DeepSee predispersion compensation. Dynamic Ca^2+^ fluorescence images (16 kHz bidirectional, ∼28 frames/s for 512 × 512-pixel scan) were acquired in real time along with electrophysiological signals of inspiratory XII nerve activities (LAS AF acquisition hardware and software electrophysiology module v.2.60). Simultaneous recording of these signals allowed us to functionally identify pre-BötC inspiratory neurons that were rhythmically active in phase with inspiratory network activity monitored by XII discharge. The infrared excitation laser for two-photon fluorescence was simultaneously used for transmission bright-field illumination to obtain a Dodt gradient contrast structural image to provide fluorescence and structural images matched to pixels. This structural imaging also allowed us to accurately place a patch pipette on functionally identified neurons in experiments using whole-cell recording. For experiments performed with GCaMP6f analyzing changes in dynamic fluorescence signals during application of pharmacological channel inhibitors (below), average fluorescence intensities (*F*) of regions of interest were quantified for each frame, and dynamic fluorescence signals (Δ*F*) were represented as running baseline (*F*_0_)–subtracted values (*F–F*_0_). We used Δ*F* not Δ*F*/*F*_0_ for the activity quantification because Δ*F* can be an order of magnitude brighter than *F*_0_ at the laser wavelength optimal for GCaMP6f (910–920 nm), and Δ*F* was not proportional to *F*_0_.

### Arterially perfused *in situ* brainstem–spinal cord preparations

To investigate contributions of TRPM4/TRPC3 channels in generation of respiratory activity in more intact systems, we also performed experiments with *in situ* arterially perfused brainstem–spinal cord preparations from mature (3–4-wk-old) rats of either sex (Sprague-Dawley, 45–100 g) or adult (3–5-mo-old) mice of either sex (C57BL/6, 25–35 g; Paton, 1996; Smith et al., 2007). Preheparinized (1000 units, given intraperitoneally) rats/mice were anaesthetized deeply with 5% isoflurane until loss of the paw withdrawal reflex, and the portion of the body caudal to the diaphragm was removed. The head and thorax were immersed in ice-chilled carbogenated ACSF solution containing the following (in mm): 1.25 MgSO_4,_ 1.25 KH_2_PO_4_, 5.0 KCl, 25 NaHCO_3_, 125 NaCl, 2.5 CaCl_2_, 10 dextrose, and 0.1785 polyethylene glycol. The brain was decerebrated at a precollicular level, and the descending aorta, thoracic phrenic nerve (PN), and cervical vagus nerves (VN) were surgically isolated. The dorsal brainstem was exposed by craniotomy and cerebellectomy. The preparation was transferred to a recording chamber and secured in a stereotaxic head frame with the dorsal side up. The descending aorta was cannulated with a double lumen catheter (DLR-4, Braintree Scientific) for ACSF perfusion with a peristaltic roller pump (505D, Watson-Marlow) and for recording of perfusion pressure with a pressure transducer. The ACSF perfusate was gassed with 95% O_2_-5% CO_2_ and maintained at 31°C. Vecuronium bromide or rocuronium bromide (2–4 µg/ml; SUN Pharmaceutical Industries) was added to the perfusate to block neuromuscular transmission. Vasopressin (200–400 pm as required; APP Pharmaceuticals) was added to the perfusate to raise and maintain perfusion pressure between 70 and 80 mmHg (Pickering and Paton, 2006).

### Electrophysiological recording *in vitro* and *in situ*

To monitor inspiratory network activity and motor output in the rhythmically active neonatal rat and mouse medullary slice preparations *in vitro*, XII motoneuron population activity was recorded from XII nerve rootlets with fire-polished glass suction electrodes (50–100-µm inner diameter). Extracellular recordings of inspiratory and post-inspiratory motoneuronal activity from VN, and inspiratory activity from PN, in the *in situ* perfused mature rat and mouse preparations were also obtained with suction electrodes (150–200-µm inner diameter). Signals in all cases were amplified (50,000–100,000×, CyberAmp 380, Molecular Devices), bandpass filtered (0.3–2 kHz), digitized (10 kHz) with an AD converter [Power Lab, AD Instruments or Cambridge Electronics Design] and then rectified and integrated digitally with Chart software (AD Instruments) for the *in vitro* slice preparations or Spike 2 software (Cambridge Electronics Design) for the perfused *in situ* preparations. Extracellular population activity from the pre-BötC in the perfused *in situ* preparations was also recorded by a dorsal approach with a fine-tipped glass pipette (3–5-MΩ resistance) filled with 0.5 m sodium acetate (Sigma-Aldrich), or in some cases with a tungsten microelectrode (3–4 MΩ), which was positioned by a computer-controlled 3D micromanipulator (MC2000, Märzhäuser). The precise location of the pre-BötC was determined by the anatomic coordinates that we initially defined by mapping neuronal activity of medullary respiratory neurons within the ventral respiratory column. The pre-BötC is readily identified by a characteristic pattern of pre-inspiratory/inspiratory population activity and is distinguishable from the more rostral BötC region, which has a characteristic profile of post-inspiratory and augmenting expiratory activities, and from the more caudal rVRG region, which has an established profile of augmenting inspiratory population activity.

For the *in vitro* slice experiments in which cytoplasm was harvested from rhythmically active pre-BötC neurons for molecular analyses (below), whole-cell current-clamp recordings were first obtained with a HEKA EPC-9 patch-clamp amplifier (HEKA Electronics) controlled by PatchMaster software (HEKA; 2.9-kHz low-pass filter, sampled at 100 kHz). Recording electrodes (borosilicate glass pipette, 4–6 MΩ), positioned with microdrives (Scientifica), contained (in mm): 130.0 K-gluconate, 5.0 Na-gluconate, 3.0 NaCl, 10.0 Hepes, 4.0 Mg-ATP, 0.3 Na-GTP, and 4.0 sodium phosphocreatine, pH 7.3 using KOH.

### Single-cell multiplex RT-PCR for mRNA expression profiling in functionally identified pre-BötC respiratory neurons

After whole-cell recording, cytoplasm of the imaged and electrophysiologically identified pre-BötC respiratory neurons was aspirated as completely as possible into the patch pipette under visual control and then immediately expelled into a thin-walled tube for PCR containing reverse-transcription reagents (Invitrogen; Koizumi et al., 2013). To avoid contamination, we continuously applied positive pressure to the autoclaved glass pipettes used for whole-cell recording as the pipette was advanced in the slice to the targeted neuron. Single-cell multiplex RT-PCR (scmRT-PCR) was subsequently performed on the cytoplasm to probe for mRNA for TRPM4 and TRPC3 channels, VgluT2, GlyT2, and glutamic acid decarboxylase 67 (GAD67) to identify channel expression in pre-BötC inspiratory glutamatergic and glycinergic/GABAergic neurons. First-strand cDNA was synthesized for 1.5 h at 50°C in a mixture of MgCl_2_ (2 µl, 25 mm), dNTPs (1 µl, 10 mm), BSA (0.7 µl, 143 ng/µl), random hexamers (1 µl, 50 ng/µl), oligodT (0.7 µl, 0.5 µg/µl), RNaseOUT (1.2 µl, 40 units/µl), DTT (1 µl, 0.1 m), and SuperScriptIII RT (1 µl, 200 units/µl). The entire reaction was either immediately used as template for multiplex PCR or frozen at –80°C until assayed. After reverse transcription, the cDNA for each target mRNA was amplified simultaneously in a multiplex PCR procedure, using primers for TRPM4 (forward, 5'-AAGCTCCCCTGCGCCATCGT-3'; reverse, 5'-AGGGCAGGCCGCGAATGGAA), TRPC3 (forward, 5'-TGGGTTCTCGGGATGATGTGGT-3'; reverse, 5'-GGGACCAGACTGAAGGGTGGAGG), VgluT2 (forward, 5'-TGTTCTGGCTTCTGGTGTCTTACGAGAG-3'; reverse, 5'-TTCCCGACAGCGTGCCAACA-3'), GlyT2 (forward, 5'-TCTGCATGACTGCCTATCCG-3'; reverse, 5'-AACACAGGCTTGTGTGTGCG-3'), and GAD67 (forward, 5'-ACCCTGGTGCCCGCTTCC-3'; reverse, 5'-TATTGGTATTGGCAGTTGATGTC-3'). The first multiplex PCR was performed as a hot-start reaction in a final volume of 50 µl containing 12 µl reverse transcription reaction, 20–50 pM of each primer, 0.2 mm dNTPs, and 10× High Fidelity PCR buffer with 2 mm MgCl_2_ and 5 U Platinum Taq High Fidelity DNA polymerase (Invitrogen). The reaction mixtures were heated to 94°C for 2 min, and 30 cycles (94°C, 30 s; 55°C, 30 s; 68°C, 1 min) of PCR were followed by a final elongation period of 10 min at 68°C. The second round of PCR amplification was performed as individual reactions with primers for TRPM4 (forward, 5'-GGCCCAAGATTGTCATCGTG-3'; reverse, 5'-TTGGCATACTGGGACACACA-3'), TRPC3 (forward, 5'-CTGCAAGCCACCAAAGCGCAC-3'; reverse, 5'-CATGTTGAGCAGAACGACCACCA-3'), VgluT2 (forward, 5'-AGGTACATAGAAGAGAGCATCGGGGAGA-3'; reverse, 5'-CAC TGTAGTTGTTGAAAGAATTTGCTTGCTC-3'), GlyT2 (forward, 5'-TCTGCATGACTGCCTATCCG-3'; reverse, 5'-CATGGTGTCAAGTCCAAGCG-3'), and GAD67 (forward, 5'-GGACTTCCACCACCCACAC-3'; reverse, 5'-CTAAACCAATGATATCCAAACCAG-3'), using 1 µl of the first PCR reaction product under similar conditions with the following modifications: 50 pM of each primer pair and 25 thermal cycles. Aliquots (10 µl) of PCR products were separated and visualized in a SYBR Green–stained agarose gel (2%) by electrophoresis. The expected sizes of PCR-generated fragments were: TRPM4 (301 bp), TRPC3 (522 bp), VgluT2 (315 bp), GlyT2 (701 bp), and GAD67 (185 bp). To ensure that the PCR signal arose from the cytoplasm of the recorded cell, the same RT-PCR assays were run on pipette solution collected from negative control “mock harvests” in each experiment (Koizumi et al., 2013). These assays were performed on the pipette solution after the pipette was advanced into the slice and withdrawn without extracting cell contents. In all scmRT-PCR assays, 100 pg total rat brain RNA (Ambion) was also run as RT template to serve as a positive control.

### Analyses of TRP channel contributions to respiratory rhythm and motor pattern generation *in vitro* and *in situ*

We performed combined electrophysiological and pharmacological experiments to probe for the contributions of endogenously active TRPM4 and TRPC3 channels to respiratory rhythm and motor pattern generation in the *in vitro* slice preparations. We analyzed the time course of perturbations of the inspiratory burst frequency, amplitude, and duration of integrated XII inspiratory motor output after application to the slice bathing solution of the putative selective TRPM4 channel inhibitor (9-phenanthrol, Millipore, 10–50 µm; Guinamard et al., 2014), the selective TRPC3 channel inhibitor (Pyrazole compound-3: Pyr3, Millipore, 10–50 µm; Kiyonaka et al., 2009), and for comparison, the putative *I*_CAN_ blocker flufenamic acid (FFA, Sigma-Aldrich, 20–75 µm; Teulon, 2000; Guinamard et al., 2013). In experiments with mouse slices expressing GCaMP6f in glutamatergic neurons, the dynamic Ca^2+^ activity within the pre-BötC (regional and single-neuron Δ*F)* was measured for 2.5-min intervals before and starting at 5, 10, 20, and 30 min after drug application to analyze local perturbations of neuronal activity within the pre-BötC rhythm-generating circuit accompanying perturbations of XII activity, which was continuously recorded throughout the experiments. Mean peak Δ*F* values computed for the 2.5-min periods were normalized to the mean peak Δ*F* values during control (before drug application) 2.5-min periods.

Contributions of TRPM4 and TRPC3 channels to respiratory rhythm and motor pattern generation in the mature rat and mouse arterially perfused brainstem–spinal cord preparations *in situ* were also analyzed by adding 9-phenanthrol (20–50 µm) and Pyr3 (50 µm) to the perfusion solution. Perturbations of the inspiratory PN motor output as well as VN inspiratory and post-inspiratory activity were analyzed. Throughout these experiments, the perfusion pressure was maintained with vasopressin added to the perfusion solution as required or by adjusting the perfusion pump speed to avoid possible effects of perfusion pressure changes on respiratory activity, since 9-phenanthrol and Pyr3 caused reductions (10–20 mmHg) in perfusion pressure, consistent with the proposed role of TRPM4 and TRPC3 channels in the control of vascular smooth muscle tone (Brayden et al., 2008).

### Signal analyses of respiratory parameters

All digitized electrophysiological signals were analyzed by automated procedures to extract respiratory parameters from integrated nerve or neuronal population activities, performed with IDL (Exelis VIS) and Matlab (R2016a, Matlab, RRID: SCR_001622) software using the NIH high-performance computing Biowulf cluster. Inspiratory events were detected from the smoothed integrated XII (*in vitro*) or PN (*in situ*) signals via a 300-ms window moving average and peak detection algorithm that calculated a threshold-based zero derivative (positive peak) point. After peak detection, inspiratory activity time (*T*_I_), expiratory interval time (*T*_E_), respiratory period (*T*_TOT_), and frequency (*f*_R_ = 1/*T*_TOT_) were computed. *T*_I_ was measured as the original integrated burst width at 20% of the peak height above baseline; *T*_E_ was calculated as *T*_TOT_ – *T*_I_. Inspiratory amplitude was calculated by subtracting the local baseline value from the peak value of the integrated signals. The endpoint of the parameter quantification was defined when inspiratory amplitude declined to either a quasi–steady state value as assessed by inspection, or to noise level with the disappearance of inspiratory activity. Representative time courses of these parameters were extracted by a 300-s-window, time-based moving median. Data were then pooled per experimental condition, and summary time courses were computed with the parameter values normalized to mean values during the control period (300–0 s before drug application).

We also quantified effects of the pharmacological manipulations on the regularity of the inspiratory rhythm by analyzing Poincaré plots of periods for 80 inspiratory bursts before and after drug application as the integrated inspiratory amplitude reached the defined endpoint. Short- and long-term period variability was quantified by plotting each *T*_TOT_ as a function of the preceding *T*_TOT_ and fitting a Gaussian distribution to these points projected onto the line perpendicular to the *y* = *x* line (with standard deviation SD1) and the points projected onto the *y* = *x* line (with standard deviation SD2). SD1 represents total burst-to-burst period variability, and SD2 represents total variability minus burst-to-burst variability, serving as measurements of short- and long-term rhythm regularity, respectively (Tulppo et al., 1996; Fishman et al., 2012). Values of SD1 and SD2 during periods of drug application were normalized to control values. Coefficients of variation of inspiratory burst periods were also calculated to measure mean normalized variability of *T*_TOT_ over the same time intervals used to determine SD1 and SD2.

For the *in situ* experiments, in addition to quantifying the respiratory parameters indicated above (*T*_TOT_, *T*_I_, *T*_E_, *f*_R_), we analyzed perturbations of amplitudes and durations of individual phases of the respiratory cycle. Amplitudes and durations of inspiratory activity in PN and VN recordings and post-inspiratory activity in VN recordings were analyzed from integrated, cycle phase–triggered neurograms aligned at the onset of inspiration defined by PN activity. Successive cycle-triggered traces were either overlaid (PN and VN separately) or represented as a dynamic raster plot to depict temporal profiles of activity before, during, and after drug application periods.

For statistical analysis (Table 1), the endpoint values, as described above, of each experiment within a group were compared with the control values with a two-sided Wilcoxon signed-rank test. Correlation analyses on data from imaging experiments were performed by computing either Pearson’s (*r*) or Spearman’s rank (*r_s_*) correlation coefficient. In all tests, significance level was set at *p* < 0.05.

**Table 1.** Summary of statistics from figures

Figure	Parameter	Type of test	*p*-value
4*A*	9-Phen: XII Amp, f_R_, T_I_	Two-sided Wilcoxon signed rank	0.031, 0.31, 0.031
4*B*	Pyr3: XII Amp, f_R_, T_I_	Two-sided Wilcoxon signed rank	0.031, 0.85, 0.062
4*C*	FFA: XII Amp, f_R_, T_I_	Two-sided Wilcoxon signed rank	0.031, 1.00, 0.031
5*A*	9-Phen: XII Amp, f_R_, T_I_	Two-sided Wilcoxon signed rank	0.031, 0.44, 0.031
5*B*	Pyr3: XII Amp, f_R_, T_I_	Two-sided Wilcoxon signed rank	0.031, 0.16, 0.031
5*C*	FFA: XII Amp, f_R_, T_I_	Two-sided Wilcoxon signed rank	0.031, 0.31, 0.062
6*B*	9-Phen: SD1, SD2	Two-sided Wilcoxon signed rank	0.31, 0.69
	Pyr3: SD1, SD2	Two-sided Wilcoxon signed rank	1.00, 0.44
	FFA: SD1, SD2	Two-sided Wilcoxon signed rank	0.31, 0.56
6*D*	9-Phen: SD1, SD2	Two-sided Wilcoxon signed rank	0.094, 0.094
	Pyr3: SD1, SD2	Two-sided Wilcoxon signed rank	0.84, 0.84
	FFA: SD1, SD2	Two-sided Wilcoxon signed rank	0.56, 0.56
8*A*	Control: f_R_, XII Amp, Δ F	Spearman’s rank correlation	0.70, 0.74, 0.62
8*B*	9-Phen: f_R_, XII Amp, Δ F	Spearman’s rank correlation	0.52, 0.00000048, 0.017
	Pyr3: f_R_, XII Amp, Δ F	Spearman’s rank correlation	0.60, 0.0011, 0.0028
	FFA: f_R_, XII Amp, Δ F	Spearman’s rank correlation	0.22, 0.00070, 0.031
8*C*	9-Phen: XII Amp vs Δ F	Pearson’s linear correlation	0.000032
	Pyr3: XII Amp vs Δ F	Pearson’s linear correlation	0.0000052
	FFA: XII Amp vs Δ F	Pearson’s linear correlation	0.0013
12*A*	9-Phen: pre-BötC, VN, PN, f_R_, T_I_	Two-sided Wilcoxon signed rank	0.031, 0.031, 0.031, 0.031, 0.44
	Pyr3: pre-BötC, VN, PN, f_R_, T_I_	Two-sided Wilcoxon signed rank	0.0078, 0.0078, 0.016, 0.016, 0.25
12*B*	9-Phen: pre-BötC, VN, PN, f_R_, T_I_	Two-sided Wilcoxon signed rank	0.031, 0.016, 0.016, 0.031, 0.031
	Pyr3: pre-BötC, VN, PN, f_R_, T_I_	Two-sided Wilcoxon signed rank	0.031, 0.031, 0.031, 0.44, 0.031

## Results

### Immunohistochemical labeling of TRPM4 and TRPC3 channels in pre-BötC neurons, regions of the ventral respiratory column adjacent to the pre-BötC, and motoneurons

**Figure 1.**
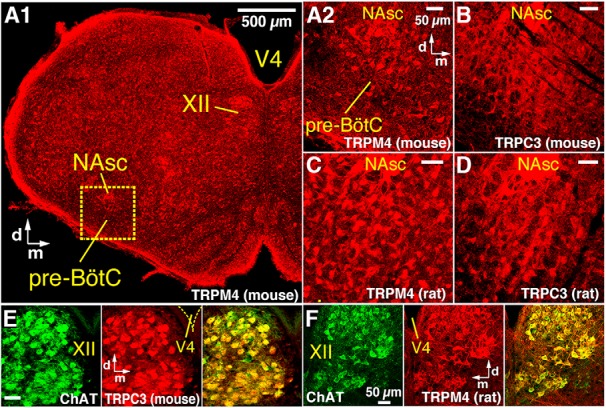
Immunolabeling of TRPM4 and TRPC3 channels in pre-BötC neurons and motoneurons. ***A***, ***B***, Confocal fluorescence microscopy images of coronal sections from neonatal mouse medulla at the level of the pre-BötC, showing widely distributed neuronal labeling by TRPM4 (***A1***, low magnification; ***A2***, higher magnification of dashed box in ***A1***) and TRPC3 (***B***) channel antibodies (red) within the pre-BötC region and motoneurons within the semicompact division of nucleus ambiguus (NAsc). Note extensive antibody labeling also outside of these regions (***A1***). ***C***, ***D***, Confocal images of the pre-BötC region from adult rats, showing neuronal labeling by TRPM4 (***C***) and TRPC3 (***D***) channel antibodies within the pre-BötC and labeling of NAsc motoneurons. ***E***, ***F***, Confocal images of the hypoglossal (XII) motor nucleus on one side of the medulla at the level containing the pre-BötC from neonatal rat (***E***) and mouse (***F***), showing TRPM4 (***E***) and TRPC3 (***F***) channel antibody labeling (red) of XII motoneurons identified by ChAT immunolabeling (green). Merged images (right in ***E*** and ***F***) show antibody colabeling of XII motoneurons. ***A–D*** have the same dorso-medial anatomic orientation. d, dorsal; m, medial; V4, 4th ventricle.

TRPM4 and TRPC3 channel antibodies labeled neurons bilaterally within the pre-BötC region (Fig. 1) in medullary slices from neonatal and mature rats/mice (*n* = 3 each). These channels were also labeled by antibody in (1) motoneurons defined by ChAT immunolabeling within nucleus ambiguus (NA) and the XII motor nucleus containing subpopulations of respiratory motoneurons; (2) neurons within the medullary reticular formation zone dorsal to pre-BötC where inspiratory XII premotor neurons are distributed (Koizumi et al., 2008; Revill et al., 2015); (3) neurons within the rostral ventral respiratory group (rVRG) region, adjacent and caudal to the pre-BötC, where bulbospinal respiratory neurons are localized; and (4) neurons in the BötC region containing respiratory neurons rostral to the pre-BötC. TRPM4 and TRPC3 channels are not exclusively expressed in these regions, but as indicated by antibody labeling are widely expressed in neurons throughout the medullary reticular formation at these levels of the medulla.

### Expression of TRPM4 and TRPC3 channels in glutamatergic and glycinergic neurons

**Figure 2.**
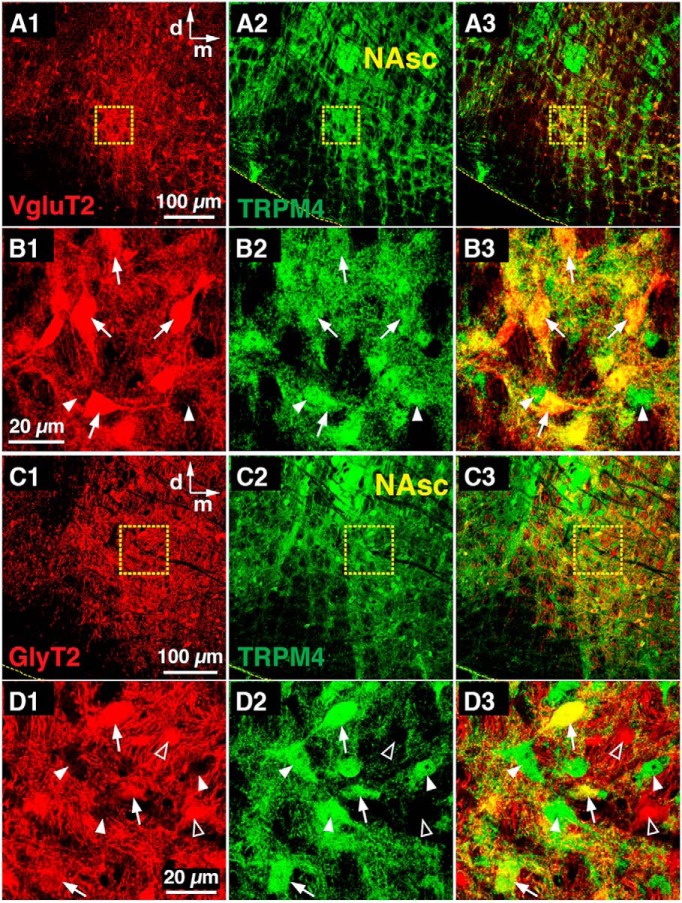
Glutamatergic and glycinergic pre-BötC neurons express TRPM4 channels. ***A***, Confocal fluorescence microscopy single plane images of a coronal section of the medulla at the level of the pre-BötC ventral to NAsc from adult VgluT2-tdTomato transgenic mouse, showing Cre-dependent tdTomato-labeled glutamatergic neurons (red, ***A1***), TRPM4 antibody-labeled neurons (green, ***A2***) throughout the pre-BötC, and their merged image (***A3***). ***B***, Single optical plane images of VgluT2 positive (***B1***) and TRPM4 (***B2***) immunolabeled neurons in the pre-BötC subregion marked by dashed box in ***A***. Merged image (***B3***) shows that a majority of VgluT2-positive pre-BötC neurons were colabeled with TRPM4 antibody (white arrows) along with TRPM4 antibody-positive, but VgluT2-negative, neurons (arrowheads). ***C***, Confocal images of the pre-BötC region from adult GlyT2-tdTomato transgenic mouse showing Cre-conditional tdTomato-labeled glycinergic neurons (red, ***C1***), TRPM4 antibody-labeled neurons (green, ***C2***), and the merged image (***C3*)**. ***D***, Single optical plane images of GlyT2-positive (***D1***) and TRPM4 immunolabeled neurons (***D2***) in the pre-BötC area marked by dashed box in ***C***. Merged image (***D3***) shows GlyT2-positive pre-BötC neurons colabeled with TRPM4 antibody (white arrows) along with TRPM4 antibody-positive, but GlyT2-negative, neurons (arrowheads) as well as TRPM4 antibody-negative, GlyT2-positive neurons (open arrowheads). All images have the same dorso-medial anatomic orientation. NAsc, semi-compact division of nucleus ambiguus; d, dorsal; m, medial.

In the transgenic mouse strains with Cre-dependent, cell type–specific expression of tdTomato fluorescent protein, we established by immunolabeling that TRPM4 and TRPC3 channels are present in both glutamatergic and glycinergic neurons within the pre-BötC, as well as other ventral medullary respiratory-related regions examined (BötC, rVRG). The majority of tdTomato-labeled pre-BötC glutamatergic neurons (73%, *n* = 1487/2049 cells) examined were colabeled by TRPM4 channel antibody in medullary sections from VgluT2-tdTomato mice (Fig. 2*A*). In sections from other VgluT2-tdTomato mice (*n* = 2), 71% (*n* = 589/826) of tdTomato-labeled pre-BötC glutamatergic neurons were labeled by TRPC3 channel antibody (not shown). We also examined immunolabeling of TRPM4 or TRPC3 channels in sets of glutamatergic neurons within the BötC and rVRG regions. The majority of tdTomato-labeled glutamatergic neurons expressed TRPM4 and TRPC3 channels in the BötC [63% (*n* = 750/1185) and 70% (*n* = 372/527) of neurons examined, respectively] and rVRG [74% (*n* = 830/1120) and 62% (*n* = 165/265)].

Because glycinergic neurons are also functional components of respiratory pattern generation circuits within the pre-BötC, BötC, and rVRG regions (Morgado-Valle et al., 2010; Richter and Smith, 2014; Shevtsova et al., 2014), we also examined immunolabeling of TRPM4 and TRPC3 channels in glycinergic neurons in these regions in GlyT2-tdTomato mice (*n* = 2 each). In the pre-BötC, 50% (*n* = 340/674) of the GlyT2-positive neurons examined were immunolabeled with TRPM4 channel antibodies (Fig. 2*B*), whereas 35% (*n* = 171/486) of GlyT2-positive neurons were labeled by TRPC3 channel antibodies. In the BötC, 67% (*n* = 633/942) and 65% (*n* = 387/587) of GlyT2-tdTomato-labeled neurons were, respectively, colabeled by TRPM4 and TRPC3 channel antibodies, whereas in the rVRG region only 27.4% (79/288) and 15.5% (31/200), respectively, of these neurons were colabeled.

### TRPM4 and TRPC3 channel mRNA in glutamatergic, glycinergic/GABAergic pre-BötC inspiratory neurons and cranial motoneurons detected by single-cell multiplex RT-PCR

**Figure 3.**
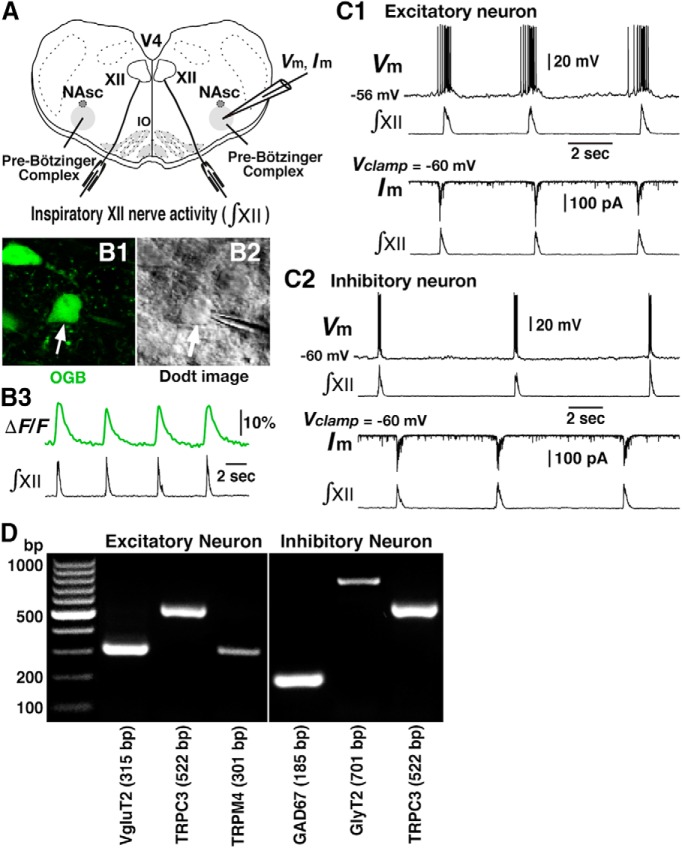
Expression of TRPM4 and TRPC3 channel mRNA in glutamatergic and glycinergic/GABAergic pre-BötC inspiratory neurons. ***A***, Overview of experimental *in vitro* neonatal rat rhythmic slice preparation showing whole-cell patch-clamp recording from the pre-BötC inspiratory neurons and suction electrode recordings from hypoglossal (XII) nerves to monitor inspiratory activity. NAsc, semicompact division of nucleus ambiguus; V4, fourth ventricle. ***B***, Two-photon single optical plane images of pre-BötC inspiratory neuron (arrow) targeted for whole-cell recording and subsequent harvesting of cytoplasm, showing Ca^2+^-sensitive dye (OGB) labeling (***B1***) and Dodt structural image (***B2***). ***B3***, Identification of inspiratory neuron by verifying that the Ca^2+^ fluorescence signals in real time are synchronized with integrated inspiratory XII nerve activity (∫ XII). ***C1***, Current-clamp recording (upper traces) from excitatory pre-BötC inspiratory neuron in ***B***illustrates inspiratory bursts synchronized with ∫ XII. Under voltage-clamp (lower traces), the same neuron exhibited rhythmic inward synaptic currents synchronized with ∫ XII. This neuron was shown to be excitatory (VgluT2-expressing) by *post hoc* single-cell RT-PCR (below). ***C2***, Current-clamp recording (upper traces) and voltage-clamp recording (lower traces) from inhibitory pre-BötC inspiratory neuron illustrating inspiratory bursts and rhythmic inward synaptic currents synchronized with ∫ XII. This neuron was shown to be inhibitory (co-expression of GlyT2 and GAD67 mRNA) by *post hoc* single-cell RT-PCR (see below). ***D***, Representative electrophoresis gel generated by single-cell multiplex RT-PCR from mRNA in cytoplasm harvested during whole-cell recording from two electrophysiologically identified pre-BötC inspiratory neurons (***C1*** and ***C2***) in neonatal rat slices. In addition to cDNA probes for TRPM4 and TRPC3 channel mRNA, probes for vesicular glutamate transporter type 2 (VgluT2), glycine transporter type 2 (GlyT2), and glutamatic acid decarboxylase 67 (GAD67) mRNA were used to identify excitatory or inhibitory neuronal phenotypes, examples of which are shown. Expected numbers of base pairs (bp) for reaction products are indicated. Assays for both of these neurons had clean negative controls from “mock harvests” in the slice and appropriate positive controls from 100 pg total rat brain RNA run as RT template (not shown, see Materials and Methods).

To confirm that respiratory neurons express TRP channels, we probed for TRPM4 and TRPC3 channel mRNA in single functionally identified pre-BötC inspiratory neurons in rhythmically active *in vitro* medullary slice preparations from WT neonatal rats and mice. Pre-BötC inspiratory neurons were identified by imaging neuronal Ca^2+^ dynamics with OGB at depths up to 150 µm in these slices, in which inspiratory neurons exhibit rhythmic Ca^2+^ fluorescence transients in phase with the inspiratory XII nerve activity (Koizumi et al., 2013). Under current-clamp recording, all optically identified pre-BötC inspiratory neurons exhibited spike discharge synchronized with rhythmic XII activity. In the cytoplasm harvested from these neurons (*n* = 41 neurons in total; *n* = 33 from 8 rat slices and *n* = 8 from 3 mouse slices) during whole-cell recording, we probed for TRPM4 and TRPC3 channel mRNA as well as VgluT2, GlyT2, and/or GAD67 mRNA to identify neuronal transmitter phenotype (Fig. 3). Only neurons with clean negative controls from “mock harvests” in the slice and appropriate positive controls (see Methods) were used for the analysis. In this sample, we identified 32 excitatory pre-BötC inspiratory neurons expressing only VgluT2 mRNA (28 neurons from rat slices; 4 neurons from mouse slices) and 9 inhibitory neurons expressing either GlyT2 mRNA only (*n* = 1 each from rat and mouse slices), GAD67 mRNA only (*n* = 1 each from rat and mouse), or coexpression of GlyT2 and GAD67 mRNA (*n* = 3 from rat and *n* = 2 from mouse slices), a phenotype previously documented for pre-BötC inhibitory interneurons (Koizumi et al., 2013). No VgluT2 mRNA was detected in these inhibitory neurons. Most of the TRP channel mRNA-positive pre-BötC inspiratory neurons in this sample were glutamatergic (*n* = 32/41, 78%), and almost half of these excitatory neurons (*n* = 15/32, 47%) coexpressed both TRPM4 and TRPC3 mRNA, while other excitatory neurons expressed either TRPM4 mRNA only (*n* = 5) or TRPC3 mRNA only (*n* = 12). Inhibitory pre-BötC inspiratory neurons also expressed TRPM4 mRNA only (*n* = 2), TRPC3 mRNA only (*n* = 5), or both TRPM4 and TRPC3 mRNA (*n* = 2).

We also analyzed expression of TRPM4 and TRPC3 channel mRNA in NA and XII motoneurons identified electrophysiologically as inspiratory motoneurons from whole-cell recording in rhythmically active slice preparations from both neonatal rats (*n* = 4) and mice (*n* = 2). We identified coexpression of TRPM4 and TRPC3 channel mRNA in all NA (*n* = 5 motoneurons in total; *n* = 2 from rats and *n* = 3 from mice) and XII (*n* = 10 total; *n* = 7 from rats and *n* = 3 from mice) inspiratory motoneurons sampled. Thus, these mRNA expression patterns are consistent with our results from immunolabeling demonstrating prominent TRPM4 and TRPC3 channel antibody labeling in all NA and XII motoneurons, and are consistent with previous results showing TRPM4 channel mRNA in laser-captured XII motoneurons (Alvares et al., 2014).

### Perturbations of inspiratory motor output *in vitro* by pharmacological inhibitors of TRPM4 and TRPC3 channels

The expression of TRPM4 and TRPC3 channel mRNA in identified pre-BötC inspiratory neurons and inspiratory cranial motoneurons, and the extensive antibody labeling of these channels in the pre-BötC region and adjacent respiratory-related regions as well as motor nuclei, suggest possible functional roles of these channels in rhythm and motor pattern generation. To test for functional endogenous activity of these channels in the rhythmically active neonatal rat and mouse slice preparations *in vitro*, we initially analyzed perturbations of the inspiratory rhythm and burst amplitude/duration of integrated XII inspiratory motor output after bath-application of the TRPM4 channel inhibitor 9-phenanthrol, the TRPC3 channel inhibitor Pyr3, and the *I*_CAN_ blocker FFA. In preliminary experiments, we determined that 9-phenanthrol (10–50 µm), Pyr3 (10–50 µm), and FFA (20–75 µm) progressively reduced the amplitude of XII inspiratory activity and, in some cases, could completely eliminate XII inspiratory motor output at 50 μM in our rat and mouse slice preparations. We therefore routinely used a single application of 50 µm for these drugs as a near upper bound for circuit activity perturbations.

**Figure 4.**
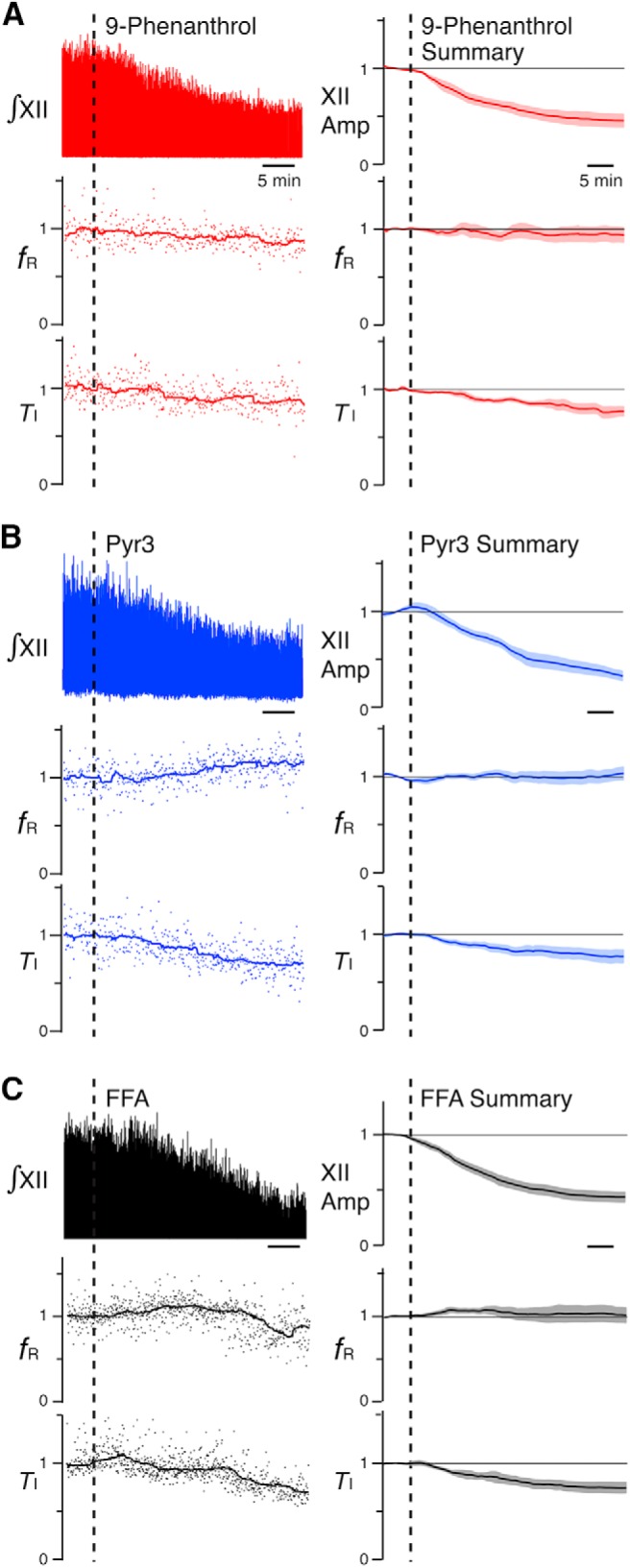
Effects of TRPM4, TRPC3, and *I*_CAN_ channel inhibitors on rhythmic hypoglossal (XII) inspiratory activity in neonatal rat medullary slice preparations *in vitro*. ***A–C***, Left panels illustrate time courses of integrated XII burst activities (∫ XII), inspiratory frequency (*f*_R_), and inspiratory activity time (*T*_I_) during application of each inhibitor (***A***, 50 µm 9-phenanthrol; ***B***, 50 µm Pyr3; ***C***, 50 µm FFA) from representative individual experiments (dots: instantaneous *f*_R_ and *T*_I_; solid lines: running median). Right panels show group summary of mean time course (solid lines) and SEM (light-color bands) of normalized integrated XII amplitude (XII Amp), *f*_R_, and *T*_I_ after drug administration (*n* = 6 slice preparations each). Time of drug administration is indicated by vertical dashed lines.

**Figure 5.**
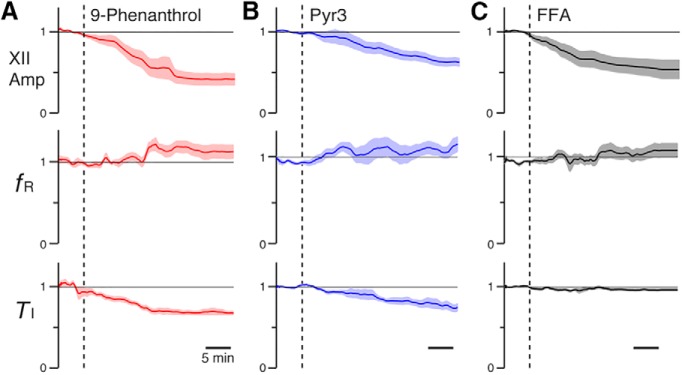
Effects of TRPM4 (***A***), TRPC3 (***B***), and *I*_CAN_ (***C***) channel inhibitors on rhythmic hypoglossal (XII) activities in neonatal mouse medullary slice preparations *in vitro*. ***A–C***, Mean time course (solid line) and SEM (light-color band) of the amplitude of normalized integrated XII burst activities (XII Amp), inspiratory frequency (*f*_R_), and inspiratory activity time (*T*_I_) during drug application (*n* = 6 slices each). Time of drug administration (***A***, 50 µm 9-phenanthrol; ***B***, 50 µm Pyr3; ***C***, 50 µm FFA) is indicated by vertical broken lines.

With 50 µm 9-phenanthrol, for both rat and mouse slices, we consistently found large perturbations of integrated XII burst amplitude without significant perturbations of inspiratory burst frequency (*f*_R_) relative to control values. Fig. 4*A* shows an example from an individual experiment, as well as the averaged, normalized time course of the reduction in XII amplitude and nonsignificant perturbation of normalized *f*_R_ as burst amplitude reached a quasi–steady state value from a set of rat slices (*n* = 6). The reduction of peak inspiratory burst amplitude for the group of experiments (57 ± 7% reduction in mean amplitude; *p* = 0.03) was accompanied by a significant reduction of inspiratory burst duration (*T*_I_, 29 ± 8% reduction; *p* = 0.03) at the defined endpoint of the time series at 32.6 ± 8.1 min. This change in amplitude and *T*_I_ was accompanied by a nonsignificant change of *f*_R_ (reduction by 6 ± 9%, *p* = 0.31) due to a small, insignificant increase in *T*_E_ (15 ± 12% increase; *p =* 0.44). Similarly, in mouse slices (*n* = 6, Fig. 5*A*) the significant reduction in peak integrated XII amplitude and *T*_I_ from control values was 60 ± 8% (*p* = 0.03) and 28 ± 3% (*p* = 0.03), respectively, without a significant change in *f*_R_ (8 ± 9% increase, *p* = 0.44) or *T*_E_ (2 ± 10% decrease, *p* = 0.69) at 20.28 ± 3.4 min after bath-application of 50 µm 9-phenanthrol. In these pharmacological experiments, as well as those described below, we obtained only partial recovery of XII burst amplitude after up to 1 h of continuous drug washout.

In both rat and mouse slices (Figs. 4*B* and 5*B*), bath-application of the TRPC3 channel inhibitor Pyr3 (50 µm) also reduced the integrated XII amplitude and *T*_I_ without significant perturbations of *f*_R_. The Pyr3-induced reduction in group (*n* = 6) mean XII amplitude and *T*_I_ in rat slices (Fig. 4*B*) was respectively 73 ± 6% (*p* = 0.03) and 27 ± 1% (*p* = 0.03), while the group mean *f*_R_ was unchanged (0 ± 7% change from control, *p* = 0.85) at the defined endpoint values at 38.8 ± 6.6 min. In mouse slices (Fig. 5*B*), the reduction in group (*n* = 6) XII mean amplitude and *T*_I_ was 47 ± 6% (*p* = 0.03) and 27 ± 6% (*p* = 0.03), while the mean *f*_R_ increased nonsignificantly above control values (20 ± 9% increase, *p* = 0.16) at the endpoint 28.2 ± 3.9 min after bath-applied 50 µm Pyr3.

Comparable data sets for perturbations of XII amplitude and *T*_I_ after bath application of the *I*_CAN_ blocker FFA (50 µm) are shown, respectively, for rat and mouse slices in Figs. 4*C* and 5*C*. FFA reduced the group (*n* = 6) mean XII amplitude and *T*_I_, respectively, in rat slices (Fig. 4*C*) by 57 ± 5% (*p* = 0.03) and 25 ± 6% (*p* = 0.03), while the mean *f*_R_ was unchanged (0 ± 10% change from control, *p* = 1.00) at the 28.3 ± 4.1-min endpoint. FFA significantly reduced the group mean (*n* = 6) XII amplitude in mouse slices (Fig. 5*C*) by 45 ± 12% (*p* = 0.03), although *T*_I_ was not significantly reduced (5 ± 1% reduction, *p* = 0.06) and *f*_R_ was nonsignificantly increased (16 ± 12%, *p* = 0.31) from control values at the quasi–steady state reached 17.1 ± 2.3 min after bath application of 50 µm FFA.

We also performed control experiments (*n* = 6 each in rats and mice), in which the XII nerve activity was recorded for 60 min without application of any pharmacological agents, and found no significant changes in inspiratory burst amplitude of XII nerve activity (100 ± 3% in rats and 99 ± 4% in mice compared with the control value at 60 min; *p* = 0.36 in rats and *p* = 0.36 in mice).

**Figure 6.**
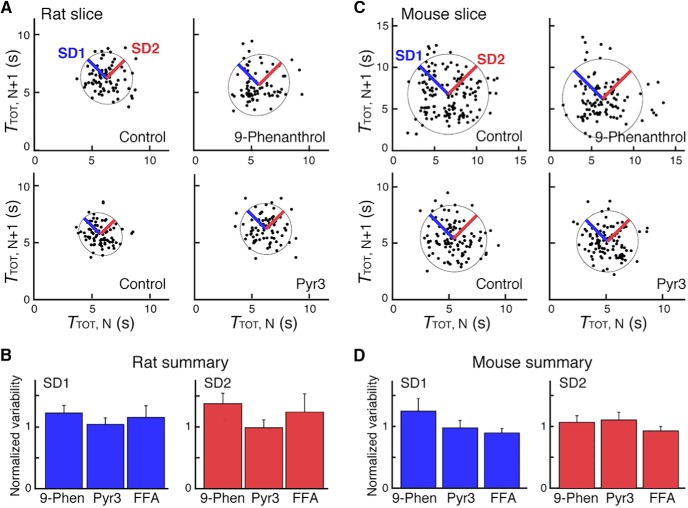
Pharmacological inhibition of TRPM4, TRPC3, and *I*_CAN_ does not affect variability of inspiratory rhythm *in vitro*. ***A***, ***B***, Poincaré maps (***A***) from a representative *in vitro* rat slice experiment with inhibition of TRPM4 and TRPC3 channels, respectively, by 9-phenanthrol (9-Phen in ***B***) and Pyr3, illustrating geometric fits of XII inspiratory period data and representations of short-term (SD1) and long-term (SD2) variability measures (see Methods for definitions), and summary data (***B***) for the analyzed group (*n* = 6 each). ***C***, ***D***, Equivalent sets of data from *in vitro* mouse slice experiments. Variability measures normalized to control values were not statistically significant in all cases in ***B*** and ***D***. *T*_TOT_, respiratory period.

In addition to establishing that 9-phenanthrol, Pyr3, and FFA did not significantly change *f*_R_ while causing large reductions in XII discharge amplitude, we also determined that these amplitude perturbations were not accompanied by significant changes in variability of the inspiratory rhythm. Short-term (burst-to-burst, SD1, Fig. 6) and longer-term (SD2) period variability, quantified for *T*_TOT_ from Poincaré maps (see Methods) for the time series analyzed as the amplitude perturbations approached quasi–steady state values for rat or mouse slices, were not significantly different from control values for each channel inhibitor (Fig. 6). For rat slices (*n* = 6), SD1 = 123 ± 12% (*p* = 0.31), 104 ± 10% (*p* = 1.00), and 116 ± 19% (*p* = 0.31) of control values for 9-phenanthrol, Pyr3, and FFA, respectively; SD2 = 138 ± 17% (*p* = 0.69), 98 ± 13% (*p* = 0.44), and 124 ± 29% (*p* = 0.56), respectively, for these inhibitors. For mouse slices (*n* = 6), SD1 = 125 ± 02% (*p* = 0.09), 97 ± 12% (*p* = 0.84), and 89 ± 7% (*p* = 0.56) of control values for 9-phenanthrol, Pyr3, and FFA, respectively; SD2 = 107 ± 11% (*p* = 0.09), 110 ± 12% (*p* = 0.84), and 93 ± 7% (*p* = 0.56) of control values for these inhibitors. Similarly, the mean coefficient of variation (CV) for the time series for each inhibitor was not significantly different from control values (Fig. 6). CV = 120 ± 12% (*p* = 0.22), 99 ± 8% (*p* = 1.00), and 120 ± 21% (*p* = 0.56) of control values for 9-phenanthrol, Pyr3, and FFA, respectively, for rat slices, and CV = 113 ± 7% (*p* = 0.16), 107 ± 10% (*p* = 1.00), and 98.0 ± 5% (*p* = 0.69) of control for 9-phenanthrol, Pyr3, and FFA, for mouse slices.

### Simultaneous perturbations within pre-BötC excitatory circuits and hypoglossal motor output *in vitro* with bath-applied channel inhibitors

**Figure 7.**
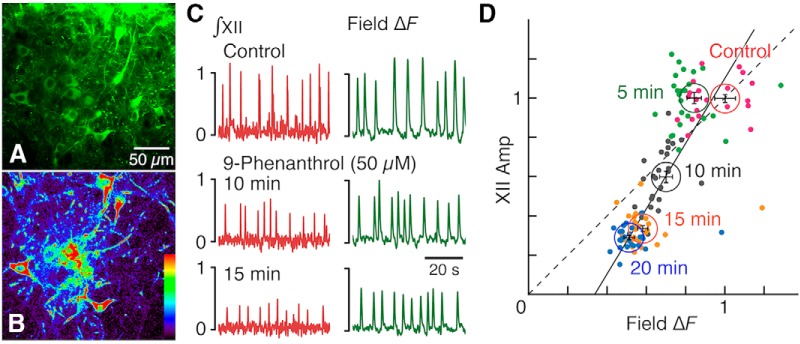
Perturbations of imaged pre-BötC inspiratory Ca^2+^ activity and electrophysiologically recorded hypoglossal motor output in the VgluT2-GCaMP6f transgenic mouse *in vitro* slice during application of TRPM4 channel inhibitor. ***A***, Example of two-photon single optical plane image showing Cre-dependent GCaMP6f expression in pre-BötC glutamatergic neurons. ***B***, Background subtracted (*F* – *F*_0_) Δ*F* image showing increased GCaMP6f fluorescence of individual neurons for the optical plane shown in ***A***. ***C***, Integrated inspiratory hypoglossal activity (∫ XII) and the spatially averaged field GCaMP6f fluorescence transients (Δ*F*), quantified as *F* – *F*_0_, of the optical plane shown in ***A*** during control time, and 10 and 15 min after bath-application of 9-phenanthrol (50 µm). ***D***, Inspiratory burst-wise correlations of the field fluorescence Δ*F* and ∫ XII amplitudes (XII Amp; colored dots), and their grouped averages (circles with error cross bars: mean values ± SEM), normalized to their control values, for time windows 5, 10, 15, and 20 min after 9-phenanthrol application. Note that the 15- and 20-min point clusters are nearly superimposed, indicating quasi–steady state of the perturbations was achieved by 20 min. The identity line (dashed) and linear regression line (solid; Pearson correlation coefficient *r* = 0.74) indicate significant correlation between peak field Δ*F* and XII Amp.

**Figure 8.**
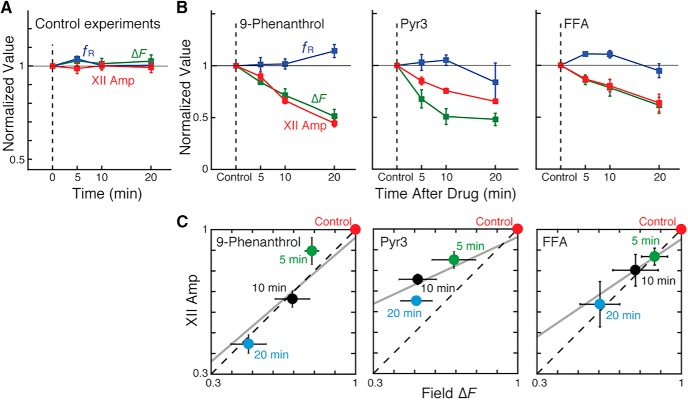
Time-dependent changes in the amplitude of integrated XII inspiratory burst activities (XII Amp) and the field GCaMP6f fluorescence transients (Δ*F*) of the pre-BötC glutamatergic population after TRPM4, TRPC3, and *I*_CAN_ inhibitors. ***A***, Control experiments (*n* = 5 mice) to test for possible photobleaching and time-dependent changes in population activity, in which calcium imaging was performed without any drug application with exactly the same protocol of image acquisition as the pharmacological experiments. The results (mean normalized values ± SEM) show that there were no significant changes in the pre-BötC field Δ*F* amplitude, XII Amp (normalized to control values), and normalized inspiratory burst frequency (*f*_R_). ***B***, Group summary data (mean normalized values ± SEM) for 9-phenanthrol, Pyr3, and FFA (*n* = 5, 5, and 4, respectively) shows reduction in both XII Amp and the pre-BötC field Δ*F* amplitude, while *f*_R_ changed nonsignificantly after applying channel inhibitors in all cases. ***C***, Time-dependent reductions of XII Amp and field Δ*F* amplitudes after drug application were positively correlated (solid lines: linear regression; Pearson linear correlation coefficient for 9-phenanthrol, Pyr3, and FFA: *r* = 0.864, 0.845, and 0.749, respectively). The linear regression on mean amplitude reduction between XII Amp and field Δ*F* for 9-phenanthrol, Pyr3, and FFA yielded corresponding linear models with slopes *m* = 0.859, 0.463, and 0.676, and intercepts *b* = 0.103, 0.499, and 0.277, respectively. Dashed lines represent the identity line.

Because TRPM4 and TRPC3 channels were expressed in pre-BötC and XII inspiratory neurons, reductions in XII activity amplitude could potentially reflect reduced excitability of XII motoneurons. To establish contributions of pre-BötC neurons, we analyzed correlations between the observed perturbations in motor output amplitude and perturbations of pre-BötC excitatory neuron population activity. For these experiments, we used *in vitro* rhythmic slices from VgluT2-GCaMP6 transgenic mice expressing the fluorescent Ca^2+^-sensor GCaMP6f in glutamatergic neurons to image neuronal and population activity within the pre-BötC (see Methods) during simultaneous recording of XII motor output (Fig. 7). Application of 50 µm 9-phenanthrol, Pyr3, or FFA to the transgenic mouse slice preparations significantly decreased the amplitude of the field Δ*F* (i.e., *F* – *F*_0_), indicating reduced excitatory neuron population activity, which typically reached quasi–steady state by 20 min after drug application (e.g., see Fig. 7*D*) and was accompanied by a significant reduction in amplitude of integrated XII activity. The time-dependent reductions of ΔF and ∫ XII amplitudes (XII Amp) were linearly correlated (see regression lines and Pearson correlation coefficients for data in Figs. 7*D* and 8*C*). The reductions in amplitude of field Δ*F* normalized to control values with 9-phenanthrol (*n* = 5), Pyr3 (*n* = 5), and FFA (*n* = 4) were 49 ± 7%, 52 ± 6%, and 39 ± 8%, respectively, at 20 min after drug administration (Fig. 8*B*). These amplitude reductions were significant over time in all cases: for 9-phenanthrol, the Spearman correlation coefficient *r_s_* = –0.88 (*p* = 0.017), for Pyr3 *r_s_* = –0.63 (*p* = 0.0028), and for FFA *r_s_* = –0.72 (*p* = 0.031). The Δ*F* amplitude perturbations were accompanied by nonsignificant changes in normalized *f*_R_ for the imaged population (Fig. 8*B*) of 14 ± 6%, –17 ± 18%, and 5 ± 6% at 20 min with 9-phenanthrol (*r_s_* = 0.14, *p* = 0.52), Pyr3 (*r_s_* = –0.11, *p* = 0.60), and FFA (*r_s_* = –0.30, *p* = 0.22), respectively. We performed control experiments (Fig. 8*A*, *n* = 5 mice) to test for possible photobleaching or time-dependent changes in population activity, in which calcium imaging was performed without any drug application with exactly the same protocol of image acquisition as the pharmacological experiments. The results showed that there were no significant changes in the pre-BötC field Δ*F* (102 ± 3% of control at 20 min, Spearman correlation coefficient *r_s_* = 0.11; *p* = 0.62), integrated XII amplitude (99 ± 3%, *r_s_* = –0.078; *p* = 0.74), and respiratory frequency (100 ± 2%, *r_s_* = 0.092; *p* = 0.70).

**Figure 9.**
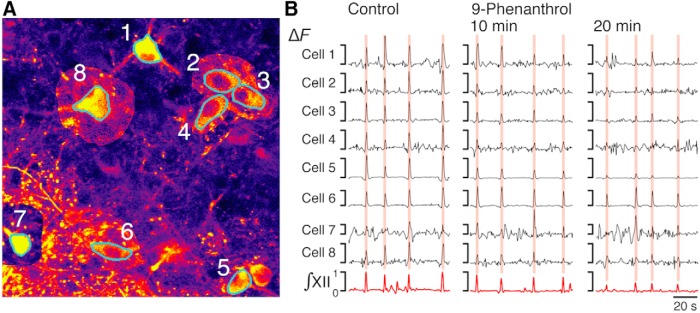
Single-neuron GCaMP6f fluorescence signal tracking during TRPM4 channel inhibition. ***A***, Single optical plane image of the pre-BötC region with cells of interest (1–8) in a rhythmically active neonatal medullary *in vitro* slice preparation from the VgluT2-GCaMP6f transgenic mouse. Regions of interest detected algorithmically (see Methods) for quantifying somal fluorescence transients are outlined in cyan. Color scale of pixels immediately surrounding some of the cells (2, 3, 7, 8) was adjusted to more clearly delineate neuron soma in this image. ***B***, Examples of time series of single-neuron GCaMPF6f fluorescence transients synchronous with integrated inspiratory hypoglossal activity (∫ XII, red) used for single-neuron Δ*F* analysis during control period, and 10 and 20 min after bath application of 50 µm 9-phenanthrol.

**Figure 10.**
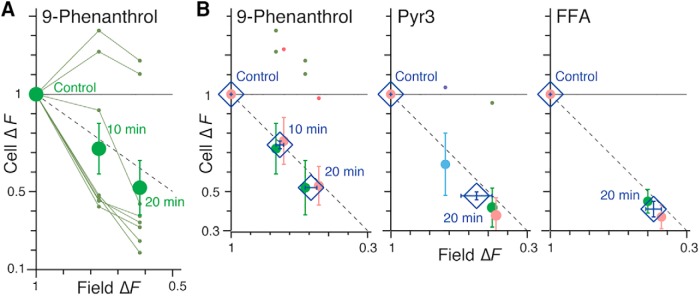
Effects of TRPM4, TRPC3, and *I*_CAN_ channel inhibitors on inspiratory Ca^2+^ activity of the pre-BötC field and glutamatergic neurons expressing GCaMP6f. ***A***, Example of relationship between normalized pre-BötC peak field GCaMP6f ΔF and normalized individual inspiratory cell ΔF, 10 and 20 min after bath-applied 50 µm 9-phenanthrol. Eight neurons (shown in Fig. 9) were tracked through time (connected green dots) within the two-photon optical section using automated ROI detection. Group mean values ± SEM of the normalized fluorescence transients are plotted (green filled circles with error bars) and the identity line (dashed) is indicated. Note that two of the neurons showed augmented fluorescence transients in this example, but the mean group cellular Δ*F* nevertheless followed the field Δ*F*. ***B***, Group summary of effects of TRPM4, TRPC3, and *I*_CAN_ inhibitors on the inspiratory pre-BötC field Δ*F* and cellular Δ*F*. Left: mean values of cellular ΔF (red, *n* = 6 neurons; green, *n* = 8, same as ***A***) ± SEM during control period, 10 min, and 20 min after bath-applied 9-phenanthrol from two slices are indicated (diamonds and error crosses). Inspiratory neurons with unaffected or increased ΔF amplitude included in the group statistics are plotted individually at the top. Middle: three-experiment summary for Pyr3 (red, *n* = 6 neurons; green, *n* = 13; blue, *n* = 4). Right: two-experiment summary for FFA (red, *n* = 12 neurons; green, *n* = 6). Identity line (dashed) is indicated.

We also tracked perturbations of Ca^2+^ transients of individual pre-BötC glutamatergic neurons (cell Δ*F*) in relation to population-level (field Δ*F*) perturbations (Figs. 9 and 10). The reduction in mean amplitude of cell Δ*F* (normalized to control amplitudes) for sets of imaged neurons with inspiratory Ca^2+^ transients was correlated with the reduction in normalized field Δ*F* over time after drug application. We note that some inspiratory neurons in these experiments exhibited normalized Δ*F* values that were unaffected or increased during drug application (outlier points in Fig. 10). Regardless, the mean cell Δ*F* for the entire group of inspiratory neurons analyzed was strongly correlated (see identity lines in Fig. 10) with the mean field Δ*F*.

### Perturbations of pre-BötC activity and motor output by TRPM4 and TRPC3 channel inhibitors in perfused brainstem–spinal cord preparations *in situ*

We analyzed contributions of endogenously active TRPM4 and TRPC3 channels to rhythm and motor pattern generation in mature rat and mouse arterially perfused brainstem–spinal cord preparations *in situ* to assess functional roles in more intact respiratory circuits generating a three-phase rhythmic activity pattern similar to that *in vivo*. We analyzed perturbations of extracellularly recorded pre-BötC and PN nerve inspiratory activities as well as VN inspiratory and post-inspiratory (post-I) activities after systemic application of 9-phenanthrol and Pyr3 in the brainstem–spinal cord via perfusion solution.

**Figure 11.**
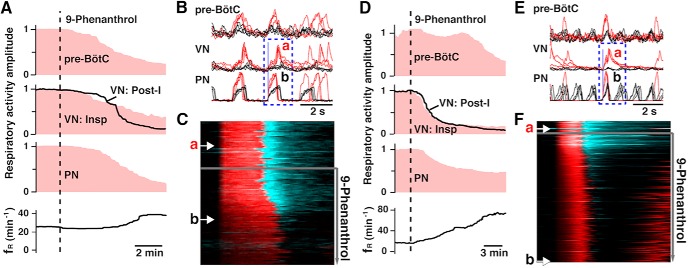
Time courses of perturbations of respiratory neural activities by TRPM4 channel inhibitor in mature rat and mouse arterially perfused *in situ* brainstem–spinal cord preparations. ***A–C***, Time courses of integrated burst amplitudes (normalized to mean control amplitudes, pink mountain plots) of inspiratory pre-BötC neural population activity (pre-BötC) obtained by extracellular recordings, vagus nerve (VN) inspiratory (Insp) and postinspiratory (post-I) activity (solid black line in middle panel in ***A***), phrenic nerve (PN) inspiratory activity, and respiratory frequency (*f*_R_) in perfused preparation from mature (4-wk-old) rat. TRPM4 inhibitor 9-phenanthrol (50 µm) was added to the perfusate at the vertical dashed line. **B,** Cycle-triggered overplots of the three neurograms (pre-BötC, VN, and PN) digitally triggered at the onset of PN activity (vertical solid line) before (red, corresponding to time points marked by arrow a in ***C***) and after the inhibitor (black traces, at arrow b in ***C***). ***C***, Dynamic raster plots of cycle-triggered PN inspiratory (red) and VN including post-I (cyan, right side) activities. After 9-phenanthrol, *T*_I_ (red) was prolonged, PN and VN inspiratory amplitude declined (darkened red), and *f*_R_ increased (see ***A***) as post-I activity amplitude declined. ***D–F***, Same type of data sets and analysis for an adult (4-mo-old) mouse preparation showing perturbations of pre-BötC, VN, and PN activity, including loss of VN post-I activity and associated increase of *f*_R_, following administration of 9-phenanthrol (20 µm).

**Figure 12.**
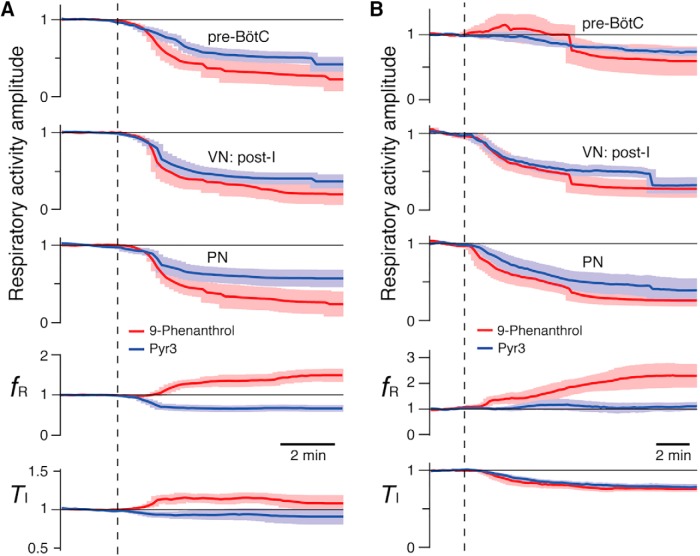
Summary of effects of TRPM4 and TRPC3 channel inhibitors on respiratory activities in arterially perfused brainstem–spinal cord preparation *in situ* from mature rats and adult mice. ***A***, ***B***, Summary time courses (solid lines: mean values; lighter color bands: ± SEM) of the amplitudes of integrated inspiratory pre-BötC neural population activity, VN post-inspiratory (post-I) activity, and PN inspiratory activity from rat (***A***, 9-phenanthrol, *n* = 6; Pyr3, *n* = 8) and mouse (***B***, 9-phenanthrol, *n* = 7; Pyr3, *n* = 6) preparations, showing significant reduction of all amplitudes (normalized to mean control values) by both TRPM4 (9-phenanthrol, red) and TRPC3 (Pyr3, blue) channel inhibitors. Bottom panels show group summaries for inspiratory frequency (*f*_R_) and inspiratory activity time (*T*_I_). Time of drug administration (9-phenanthrol, 20–50 µm; Pyr3, 50 µm) is indicated by vertical dashed lines.

Perturbations of respiratory pattern and frequency caused by 9-phenanthrol are illustrated by examples in Fig. 11 for rat (Fig. 11*A–C*) and mouse (Fig. 11*D–F*) preparations, which show time courses of the perturbations of normalized amplitudes of integrated PN and VN respiratory motor output and simultaneously recorded pre-BötC inspiratory population activity, and *f*_R_ by PN. Mean normalized time courses summarizing the perturbations by 9-phenanthrol and Pyr3 for the groups of rat and mouse preparations analyzed are presented in Fig. 12. In general, these data sets show that systemic administration of 9-phenanthrol (50 μm for *n* = 6 rat preparations, and 20–50 μm (pooled) for *n* = 7 mouse preparations) reduced the quasi–steady state amplitudes (analyzed at 8 and 14 min, respectively, for rat and mouse preparations) of integrated inspiratory activity of the pre-BötC (rat: 78 ± 16% decrease, *p* = 0.03; mouse: 39 ± 21% decrease, *p* = 0.03), and PN (rat: 77 ± 16% decrease, *p* = 0.03; mouse: 74 ± 7% decrease, *p* = 0.016). The amplitude of integrated VN inspiratory activity (e.g., Fig. 11) and post-I activity was also strongly reduced (rat post-I: 81 ± 14% decrease, *p* = 0.03; mouse post-I: 62 ± 11% decrease, *p* = 0.02). Mean normalized inspiratory *f*_R_ increased significantly after application of 50 µm 9-phenanthrol in both rats and mice (by 49 ± 15%, *p* = 0.03, and 129 ± 43%, *p* = 0.03, respectively), due to a reduction in expiratory phase duration (*T*_E_) in the rat *in situ* preparations by 44.5 ± 8.5% (*p* = 0.03) and mouse preparations by 57 ± 2.4% (*p* = 0.03), accompanied by relatively small perturbations of *T*_I_ (rat: 8 ± 11% increase, *p* = 0.44; mouse: 24 ± 3% decrease, *p* = 0.03).

Pyr3 (50 μM, *n* = 8 rat and 6 mouse preparations) reduced the normalized amplitudes of integrated inspiratory activity of the pre-BötC (rat: 58 ± 9% reduction, *p* = 0.01; mouse: 26 ± 7% reduction, *p* = 0.03), and PN (rat: 43 ± 11% reduction, *p* = 0.02; mouse: 61 ± 15%, *p* = 0.03; Fig. 12). The amplitude of integrated VN post-I activity was reduced by 64 ± 9% (*p* = 0.01) and 69 ± 10% (*p* = 0.03) in rat and mouse preparations, respectively. These perturbations were accompanied by a decrease in the group mean *f*_R_ by 33 ± 8% (*p* = 0.01) from control values in rat preparations, but no significant perturbations of *f*_R_ in mouse preparations (10 ± 14% increase, *p* = 0.44).

## Discussion

Biophysical mechanisms generating and transmitting rhythmic activity within excitatory pre-BötC circuits remain undefined despite extensive experimental and modeling studies investigating possible Na^+^- and Ca^2+^-based mechanisms of rhythmic pre-BötC cellular and population-level bursting activity (Rybak et al., 2014). Ca^2+^ signaling–based theories incorporating *I*_CAN_, postulated to be mediated by Ca^2+^-activated TRPM4 channels (Mironov, 2008; Del Negro et al., 2010), have been proposed. However, TRPM4 and other potentially important TRP channels mediating nonselective cationic currents involved in Ca^2+^-related signaling such as TRPC3, although proposed, have not been identified in pre-BötC and other respiratory neurons. Furthermore, their functional roles in respiratory circuits have not been clearly defined.

We obtained evidence for TRPM4 and TRPC3 channel mRNA in pre-BötC inspiratory neurons as well as medullary respiratory motoneurons. The pharmacologically induced perturbations of circuit activity by their putative selective channel inhibitors indicate endogenous activity of these channels with major functional roles in formation of inspiratory and post-inspiratory respiratory activity. However, our results indicate that these channels do not contribute to the generation and stability of inspiratory rhythm in pre-BötC circuits. These results therefore do not support previous Ca^2+^ signaling-based hypotheses incorporating TRPM4/*I*_CAN_ in pre-BötC neurons as a fundamental mechanism for rhythm generation.

### TRPM4 and TRPC3 channels in respiratory neurons

Our initial survey of neuronal expression of TRPM4 and TRPC3 channels by antibody labeling in neonatal/adult rats and mice, including in genetically specified excitatory and inhibitory neurons in mice, established that these channels are present in neurons in the pre-BötC region and adjacent medullary respiratory-related regions as well as in motor nuclei known to contain respiratory neurons. We assayed for TRPM4 and TRPC3 channel mRNA in identified inspiratory pre-BötC neurons and motoneurons, which has previously not been performed, although the presence of these channels has been suggested by TRPM4/5 mRNA or TRPC3/C7 channel protein detection in bulk tissue obtained from the pre-BötC region (Crowder et al., 2007; Ben-Mabrouk and Tryba, 2010). Similarly, TRPM4 channel mRNA has been detected in laser-captured XII rat motoneurons (Alvares et al., 2014), and immunolabeling of TRPM4 channel protein in mouse NA has been reported (Del Negro et al., 2010). These approaches have not specifically established channel expression in respiratory neurons.

Our scmRT-PCR and immunohistological analyses identified TRPM4 and/or TRPC3 mRNA in both excitatory and inhibitory pre-BötC inspiratory neurons in slices from neonatal rats and mice. Coexpression of mRNA for these two channels in single pre-BötC inspiratory neurons was found in nearly half of the excitatory neurons. Our sample of inspiratory pre-BötC inhibitory neurons was not sufficient to allow conclusions about channel coexpression in these neurons. Although TRPM5 mRNA (Crowder et al., 2007) and TRPC7 channel protein (Ben-Mabrouk and Tryba, 2010) in the pre-BötC region has been reported, we did not systematically probe for other TRPM/C channels in our sample of pre-BötC inspiratory neurons. Consistent with our immunolabeling results, we also found mRNA for TRPM4 and TRPC3 channels in functionally identified XII and NA inspiratory motoneurons. In general, our findings imply that TRPM4/C3 channels may be functionally involved at multiple levels within medullary respiratory circuits.

### Role of TRPM4 and TRPC3 channels in respiratory pattern generation *in vitro*

We analyzed functional contributions of TRPM4 and TRPC3 channels with the selective pharmacological inhibitors 9-phenanthrol and Pyr3, respectively, initially within rhythmically active *in vitro* slices from neonatal rats and mice. Perturbations of circuit activity were compared with those caused by FFA, a blocker of *I*_CAN_ and TRPM4 (Guinamard et al., 2013), that has been used previously to evaluate roles of this current in generating rhythmic bursting activity of respiratory neurons and circuits *in vitro* (e.g., Peña et al., 2004; Del Negro et al., 2005). We empirically determined from initial *in vitro* studies and subsequently used concentrations (maximally 50 µm for the data presented) of 9-phenanthrol, Pyr3, and FFA that produced large or near-maximal perturbations of the amplitude of inspiratory motor outputs *in vitro*, and are also expected to be effective and relatively selective for TRPM4, TRPC3, and *I*_CAN_ in physiologic preparations (Kiyonaka et al., 2009; Guinamard et al., 2013, 2014). The three channel inhibitors similarly reduced the amplitude of XII inspiratory motoneuronal activity *in vitro* without significant perturbations of inspiratory frequency and regularity of the rhythm. These amplitude perturbations indicate that currents inhibited by 9-phenanthrol, Pyr3, or FFA are endogenously active in respiratory neurons *in vitro*. The similar perturbations with 9-phenanthrol and FFA are consistent with the proposal from pharmacological studies in other cells and tissues that FFA blocks TRPM4 channels (Guinamard et al., 2014), as well as the proposal that TRPM4, known to be directly activated by increases in intracellular Ca^2+^, mediates an *I_CAN_* (Launay et al., 2002; Hofmann et al., 2003) active in respiratory neurons.

In previous pharmacological studies investigating the role of *I_CAN_* in active respiratory circuits in neonatal mouse slices *in vitro* (Peña et al., 2004), high concentrations of FFA (500 µm) reduced the amplitude/area of integrated inspiratory population activity but caused a relatively small (∼20%) reduction of inspiratory burst frequency without affecting regularity of the rhythm. Although these high concentrations of FFA can also depress voltage-gated Na^+^ currents (at >100 µm in hippocampal neurons; Yau et al., 2010) and voltage-gated Ca^2+^ currents (Shimamura et al., 2002), as well as cause other nonselective perturbations of neuronal excitability (Guinamard et al., 2013), these observations are generally consistent with our results with FFA in both neonatal rat and mouse *in vitro* slices showing relatively large perturbations of the amplitude of pre-BötC and XII motoneuronal inspiratory activity and only small perturbations of inspiratory frequency without affecting stability of the inspiratory rhythm. In other earlier *in vitro* mouse slice experiments, 100 µm FFA reduced inspiratory drive potentials of individual pre-BötC neurons, but without reducing the amplitude or altering the frequency of XII motor discharge (Del Negro et al., 2005; Pace et al., 2007). The lack of perturbations of XII inspiratory activity at this concentration of FFA is difficult to reconcile with our electrophysiological and Ca^2+^ imaging results showing correlations between the amplitude of pre-BötC inspiratory activity, as assessed by intracellular Ca*^2+^* dynamics (below), and the reduction of XII inspiratory discharge amplitude. In those earlier studies, however, higher concentrations (300–350 µm) of FFA that are considered nonselective for *I*_CAN_ reduced the XII inspiratory activity amplitude and could have eliminated rhythmic inspiratory XII motor output.

Consistent with our scmRT-PCR results indicating TRPC3 channel mRNA expression in identified inspiratory neurons, the amplitude perturbations produced by Pyr3 suggest that TRPC3 channels are also functionally activated during respiratory circuit activity *in vitro*. These channels are not directly Ca^2+^ activated, but mediate Na^+^/Ca^2+^ currents, and may be involved in regulating neuronal Ca^2+^-related signaling (Talavera et al., 2008; Birnbaumer, 2009; Guinamard et al., 2013) in respiratory neurons. This TRPC3 channel-mediated Ca^2+^ flux/intracellular Ca^2+^ regulation can potentially also affect TRPM4/*I*_CAN_ in respiratory neurons. We found coexpression of mRNA for TRPC3 and TRPM4 channels in approximately half of the excitatory pre-BötC inspiratory neurons and all of the respiratory motoneurons assayed, suggesting that such a functional interaction may be possible. The roles of TRPC3-mediated cationic currents/Ca^2+^-related signaling in generating respiratory neuron activity have not been previously investigated. Our results suggest an important functional role of these channels in activity amplitude modulation but not in rhythmogenesis, like TRPM4/*I*_CAN_ channels. Whether the similar amplitude perturbations by the TRPC3 and TRPM4 channel inhibitors reflects involvement with TRPM4/*I*_CAN_ activation by Ca^2+^-related functions of TRPC3 channels remains to be determined.

In general, the optimal pharmacological strategy for probing the roles of TRPM4/*I*_CAN_ or TRPC3 channels active in respiratory circuit neurons has not been definitively established. The major problem is to identify and analyze the neuronal currents attenuated by the channel inhibitors in respiratory neurons at any applied concentrations. Measurements of whole-cell currents mediated by TRPM4/*I*_CAN_ or TRPC3 channels in respiratory neurons have not yet been performed, so that 9-phenanthrol and Pyr3 concentrations likely to be effective/selective have been inferred in part from pharmacological analyses performed in other (typically nonneuronal) cell types (Kiyonaka et al., 2009; Guinamard et al., 2014). The problem is particularly complicated for resolving the Ca^2+^-activated TRPM4/*I*_CAN_-mediated currents, because the sources of Ca^2+^ flux activating these currents in respiratory neurons need to be preserved and taken into account in a detailed pharmacological analysis of currents activated endogenously during respiratory neuronal activity. Although Ca^2+^ flux through voltage-gated Ca^2+^ channels (Peña et al., 2004; Morgado-Valle et al., 2008), and/or synaptically activated Ca^2+^-fluxes, including through ionotropic glutamatergic receptors and/or activation of metabotropic glutamatergic receptors to induce ER Ca^2+^ release, have been postulated to activate *I*_CAN_ (Pace et al., 2007; Mironov, 2008; Del Negro et al., 2010), these mechanisms have not been established.

### Correlated perturbations of pre-BötC excitatory neuronal population activity and hypoglossal motor output *in vitro*

We initially evaluated roles of TRPM4 and TRPC3 channels by analyzing perturbations of XII inspiratory motor output in slices, but this approach does not necessary allow assessment of the contributions of these channels in pre-BötC neurons to perturbations of the motor output, since TRPM4 and TRPC3 channels are also expressed in XII inspiratory neurons. We also note that TRPM4 or TRPC3 channel antibody labeling was identified in regions of the reticular formation dorsal to the pre-BötC region that contains inspiratory XII premotoneurons (Koizumi et al., 2008; Revill et al., 2015). Accordingly, reductions in XII activity amplitude by channel inhibitors in our *in vitro* slice preparations potentially reflect reduced activity of XII motoneurons and possibly other neurons within inspiratory drive transmission circuits. We therefore more directly established involvement of pre-BötC neurons by dynamic Ca^2+^ imaging of inspiratory pre-BötC neuronal activity in slices from transgenic mice expressing GCaMP6f in glutamatergic neurons during bath application of the channel inhibitors. This allowed us to assess activity perturbations of the critical populations of pre-BötC excitatory neurons generating inspiratory rhythm and synaptic drive in transmission circuits to XII motoneurons, and to compare simultaneous perturbations of activity of these neurons and XII motor output. All of the channel inhibitors caused reductions in the amplitude of spatially averaged GCaMP6f fluorescence transients (field Δ*F*) and the fluorescence transients of individual glutamatergic inspiratory pre-BötC neurons (cell Δ*F*), confirming inspiratory activity perturbations at the level of pre-BötC excitatory neurons. Furthermore, we established that the field Δ*F* reflects the mean cell Δ*F* of sets of individual excitatory inspiratory neurons. We also found significantly correlated, linear relationships (Fig. 8) between the amplitude of field Δ*F* and the amplitude of integrated XII inspiratory activity. The slopes of these linear relationships for 9-phenanthrol, FFA, and Pyr3 (0.86, 0.68, and 0.46, respectively), particularly with Pyr3, reflect that initially the activity amplitude perturbations of our imaged sample of inspiratory glutamatergic neurons tended to occur more rapidly than the reduction in integrated XII activity, but the group mean amplitude perturbations tended to converge toward the identity line of the field Δ*F* versus integrated XII amplitude relationships as the quasi–steady-state perturbations were reached. Our results suggest that the number of active pre-BötC inspiratory cells and the burst amplitude of each active pre-BötC neuron after application of the channel inhibitors are important factors contributing to the overall reduction of pre-BötC field Δ*F* and decrease of XII burst amplitude.

We conclude that the perturbations of pre-BötC inspiratory activity correlate with the perturbation of XII inspiratory activity amplitude. The Δ*F* amplitude perturbations occurred without significant changes in the frequency of inspiratory-related activity within the pre-BötC and simultaneously recorded XII inspiratory activity. The extent to which activity perturbations of XII inspiratory motoneurons or neurons within the rhythmic inspiratory drive transmission premotor circuits contribute to the reduction of XII motor output remains to be determined. We also note that there is a subpopulation of pre-BötC inspiratory neurons with axonal projections to XII motoneurons (Koizumi et al., 2008, 2013), and reduced activity of these neurons may also contribute to the overall reduction of inspiratory activity in the transmission circuits without perturbing rhythm generation.

### Contributions of TRPM4 and TRPC3 channels to respiratory pattern generation in mature rodent brainstem circuits *in situ*

We also analyzed functional contributions of TRPM4 and TRPC3 channels with 9-phenanthrol and Pyr3, respectively, in mature rat and mouse arterially perfused *in situ* brainstem–spinal cord preparations to evaluate roles of these channels in more intact circuits generating a eupneic-like three-phase respiratory motor output pattern. Moreover, extending our analysis to mature animals was necessary, since *I*_CAN_-dependent neuronal bursting, and accordingly potential involvement of TRPM4, has been proposed to contribute to inspiratory rhythm generation predominantly in mice older than P5 (Peña et al., 2004; Del Negro et al., 2005), although *I*_CAN_ is postulated to contribute to formation of drive potentials generating inspiratory bursts throughout development (Del Negro et al., 2005). In agreement with our results obtained *in vitro*, presumptive inhibition of TRPM4 or TRPC3 channels in the more intact rat and mouse circuits *in situ* significantly reduced the amplitude of pre-BötC inspiratory activity, accompanied by reduced amplitudes of inspiratory motor outputs as evaluated from integrated vagal and phrenic nerve inspiratory activities. In addition, the channel inhibitors, especially 9-phenanthrol, caused large reductions in vagal post-I activity (e.g., Fig. 11), indicating an important contribution of endogenous channel activation to inspiratory-expiratory pattern generation in more intact respiratory circuits. The increase of respiratory frequency, primarily with TRPM4 channel inhibition, occurred with the reduction of expiratory phase duration as post-I activity was reduced (Smith et al., 2007), although rhythm generation was not disrupted.

The reduction of post-I vagal activity could reflect contributions of TRPM4 and TRPC3 channel activation at the level of vagal motoneurons, or at the interneuronal level in excitatory/inhibitory circuits in ventral medullary respiratory-related regions, including within the BötC, that generate post-I activity (Smith et al., 2007; Richter and Smith, 2014) and where these channels may be present in excitatory/inhibitory neurons as suggested by our immunolabeling results. According to the respiratory central pattern generation (CPG) network model based on experimental analyses with *in situ* preparations (Rubin et al., 2009b), different types (e.g., neurotransmitter phenotypes, active phase, bursting pattern) of respiratory interneurons in the pre-BötC and BötC are functionally interacting to generate a normal three-phase pattern of respiratory neural activity. Our experimental results of different effects of TRPM4 or TRPC3 channel inhibition on *f*_R_ in the more intact *in situ* preparations (Fig. 12) suggest different sensitivity to TRPM4 or TRPC3 channel inhibitors among different types of respiratory neurons in the CPG circuits. Contributions of TRPM4 and TRPC3 channel activation in different types of excitatory and/or inhibitory respiratory neurons remain to be clarified.

Our results suggest that inhibiting TRPM4/*I*_CAN_ or TRPC3 in excitatory pre-BötC inspiratory neurons primarily contributes to the amplitude decrease of inspiratory motor outputs *in vitro* and *in situ*, whereas inhibiting TRPM4 or TRPC3 channels in inhibitory neurons, possibly BötC expiratory neurons, in the more intact *in situ* circuits causes perturbations of post-I activity (e.g., Marchenko et al., 2016) and therefore respiratory frequency. In summary, we suggest that endogenous activation of TRPM4/*I*_CAN_ or TRPC3 plays an important role in regulating activity of excitatory and inhibitory respiratory neurons, the latter particularly in the intact *in situ* CPG circuits for inspiratory-expiratory pattern generation.

### Implications for proposed *I*_CAN_-dependent and other mechanisms of respiratory rhythm generation in pre-BötC circuits

Based on previous experimental studies in neonatal mouse rhythmic medullary slices *in vitro* (Crowder et al., 2007; Pace et al., 2007; Pace and Del Negro, 2008) and organotypic pre-BötC cultures (Mironov, 2008, 2013), and also computational modeling studies (Rubin et al., 2009a; Dunmyre et al., 2011), an emergent *I*_CAN_-dependent mechanism in pre-BötC excitatory circuits was postulated to play a major role in respiratory rhythmogenesis according to the “group pacemaker” hypothesis (Feldman and Del Negro, 2006; Del Negro et al., 2010). In this model, synaptically-activated Ca^2+^ fluxes, especially mediated by metabotropic glutamate receptors (mGluRs), were proposed to trigger *I*_CAN_ activation through intracellular Ca^2+^ signaling involving inositol triphosphate (IP_3_)-mediated Ca^2+^ release from ER stores. Activation of *I*_CAN_ is proposed to generate depolarization of excitatory inspiratory pre-BötC neurons to primarily produce synaptically mediated (i.e., network-dependent) inspiratory drive potentials underlying inspiratory bursts (Crowder et al., 2007; Pace et al., 2007; Pace and Del Negro, 2008). During population-level inspiratory bursts, the *I*_CAN_-dependent depolarization has been suggested to cause partial voltage-dependent inactivation of neuronal spike-generating transient Na^+^ channels, associated with transient depression of recurrent excitation and circuit-generated excitatory synaptic drive to deactivate *I*_CAN_ and terminate inspiratory bursts (Rubin et al., 2009a; Del Negro et al., 2010). In other more complex models with multiple sources of neuronal Ca^2+^ flux (Jasinski et al., 2013; Rybak et al., 2014), including voltage-gated Ca^2+^ currents, it has been theoretically shown that *I*_CAN_-induced bursting, and subsequent burst termination sufficient for rhythmogenesis, can occur by dynamic Ca^2+^-dependent activation-inactivation of IP_3_ receptor-mediated Ca^2+^ release, without or with involvement of other burst-terminating mechanisms such as Na^+^/K^+^ pump currents. The Na^+^/K^+^ pump currents can hypothetically contribute importantly to inspiratory burst termination and may be regulated by *I*_CAN_-mediated Na^+^ flux, linking *I*_CAN_ activation to another mechanism for inspiratory burst termination critical for rhythm generation. Interfering with ER Ca^2+^ release mechanisms does not disturb inspiratory rhythm generation in the pre-BötC *in vitro*, however, indicating that normally this source of Ca^2+^ flux is not critically involved in rhythm generation or control of inspiratory amplitude *in vitro* (Beltran-Parrazal et al., 2012), so other Ca^2+^ sources explored in these models seem to be involved in activating *I*_CAN_.

Another important hypothesis for inspiratory rhythm generation long proposed in the field is that *I*_CAN_-dependent, FFA-sensitive pre-BötC inspiratory pacemaker neurons (i.e., inspiratory neurons with intrinsic oscillatory bursting properties when isolated from synaptic inputs), with *I*_CAN_ activation driven by voltage-gated, Cd^+2^-sensitive Ca^2+^ currents, have a critical rhythmogenic role (Peña et al., 2004) in pre-BötC circuits together with other populations of neurons with oscillatory bursting properties mediated by persistent Na^+^ current (*I*_NaP_; dual pacemaker hypothesis; Thoby-Brisson and Ramirez, 2001; Peña et al., 2004; Ramirez et al., 2011), following the proposal of *I*_NaP_-dependent cellular and excitatory population rhythm generation mechanisms in pre-BötC excitatory circuits (Butera et al., 1999a, b).

In general, our results do not support the concept that populations of pre-BötC neurons with *I*_CAN_-mediated bursting properties are critically involved in generating inspiratory rhythm. However, they support the proposal that TPRM4/*I*_CAN_-mediated currents are functionally active in respiratory neurons and importantly contribute to inspiratory burst generation determining the amplitude of pre-BötC neuronal population activity. TRPC3 channels also have this fundamental role, possibly by providing Ca^2+^ flux activating TRPM4/*I*_CAN_. Remarkably, this amplitude control is essentially independent of the inspiratory rhythm generation mechanism and indicates there is a rhythmogenic kernel subpopulation of neurons within the pre-BötC excitatory network that rely on a fundamentally different oscillatory mechanism. Previous studies have proposed (Butera et al., 1999a, b) and presented evidence (Koizumi and Smith, 2008) that *I*_NaP_-dependent mechanisms are sufficient to account for a number of features of inspiratory rhythm generation when neonatal pre-BötC circuits are isolated *in vitro,* as well as in reduced *in situ* preparations from mature rats (Smith et al., 2007). In the more intact mature system, this *I*_NaP_-dependent oscillatory mechanism may not be sufficient to explain rhythm generation, which involves more complex sets of inhibitory circuit interactions with the pre-BötC excitatory circuits (Smith et al., 2007; Rubin et al., 2009b). The present studies indicate that although TPRM4/*I*_CAN_-mediated currents are functionally active in the more intact system and have a basic role in inspiratory-expiratory respiratory pattern generation, they are also not essential for rhythm generation.

## Synthesis

Reviewing Editor: Douglas Bayliss, University of Virginia School of Medicine Decisions are customarily a result of the Reviewing Editor and the peer reviewers coming together and discussing their recommendations until a consensus is reached. When revisions are invited, a fact-based synthesis statement explaining their decision and outlining what is needed to prepare a revision will be listed below.

This manuscript was seen by two reviewers, both of whom were positive about the results and the importance of the findings for the field. That said, each raised a number of concerns that are provided below. The majority of these can be addressed by revising the manuscript for further detail and better clarity. When preparing a revised manuscript, please provide examples of whole cell recordings from the pre Bötzinger cell types used for PCR, preferably also illustrating effects of the different blockers on those cells (and on hypoglossal motoneurons). In terms of additional control experiments, some assurance that decreases in calcium fluorescence or nerve activity are due to drug-associated effects on the channels (rather than photobleaching or some time-dependent technical artifact) should be provided. Finally, please provide more information on antibody specificity; the simple reference to “no primary” controls is inadequate for reagent validation.

Reviewer #1

Advances the Field

This report provided some evidences that TRPM4 and TRPC3 could be involved in the inspiratory burst generation.

Comments to the Authors

This report provided some evidences that TRPM4 and TRPC3 could be involved in the inspiratory burst generation. This study was well designed and documented. Although results are rather clear and interesting, further explanations would be needed in some parts and some discussion seems to be missed. My comments are below.

Introduction

Page 4, para 2- Page 5: Authors actually mentioned results here with some discussion. The description seems to be too much for the introduction and to include those that may be not very conclusive. Authors should rewrite these parts more concisely as suitable for introduction.

Methods

Page 11, para 2: Regarding extracellular population activity from the pre-BötC in the perfused in situ preparation, was it performed by the dorsal approach? How did you determine the precise region of the pre-BötC?

Results

Page 25, para 1, the last part: Authors described non-significant changes in normalized fR after application of antagonists. However, data of Fig. 9 showed apparent decrease of fR. Was this example not typical?

Page 26, para 3, “yet less than non-specific concentrations of 9-phenanthrol”: What was non-specific concentration? What responses were induced if you use non-specific concentration?

Discussion

Results from RT-PCR of identified neurons showed fairly small number of inhibitory pre-BötC neurons were TRPM4 and/or TRPC3 positive, suggesting that many inhibitory neurons are TRPM4 and/or TRPC3 negative, i.e. directly insensitive to the channel antagonists (is it correct?). Do you suggest any functional role of these inhibitory inspiratory neurons? These inhibitory neurons may contribute to perturbation of burst activity during application of the channel blockers (by being directly not sensitive to the drugs).

Although authors discussed some difficulty of detailed mechanisms of contribution of TRPM4/ICAN or TRPC3 on the inspiratory burst generation, analyses of electrophysiological properties (spike frequency, drive potential, etc.) obtained by whole-cell recordings of inspiratory neurons might be helpful for understanding the inhibitory effects.

What is a main factor (e.g. number of active cells, burst amplitude of each active cells or others) for decrease of XII burst amplitude and field deltaF amplitude of calcium signal in the pre-BötC by channel antagonists?

Pyr3 induced a decrease in fR in rat in situ preparation. Please discuss about this result.

Figures

Fig. 1 and 2: Although I believe that these regions are in the pre-BötC, some atlas (with lower magnification?) indicating landmarks of the regions would be helpful for better understanding of general readers who are not familiar in this field.

Fig. 2: Why is tdTomato expressed by ‘green’? (‘Red’ is more natural). Please add some explanations or show also gray scale figures (?).

Fig. 3: More delated explanations for this figure are needed; column labels, maker labels, etc. I understood that the cytoplasm was treated separately for RT-PCR to identify five types of molecules as indicated in each column. Is it OK?

There is no example of the whole-cell recording from inspiratory neurons in any figures. Indication of some examples somewhere (e.g. in Fig. 3) with recording sites might be better.

Reviewer #2

Comments to the Authors

The present study investigates the potential role of TRPM4, a putative molecular substrate for the calcium-activated non-selective cation current ICAN, and TRPC3, involved in regulating calcium-related signaling in neurons, in contributing in respiratory pattern and/or rhythmogenesis. Using a combination of electrophysiological neuronal activities recordings, calcium imaging, immunostainings and single-cell multiplex RT-PCR performed on different preparations (in vitro brainstem slice preparations, arterially perfused in situ brainstem-spinal cord preparations) and different animal models (rats or mice) the authors showed that pharmacological blockade of ICAN by different agents targeting either TRPM4 or TRPC3 channels or the ICAN current itself, leads to a reduction of the amplitude of inspiratory interneuronal (preBötC neurons) and motoneuronal (hypoglossal motoneurons) activity without significantly affecting the frequency of the fictive inspiratory rhythm. The authors conclude that activation of TRPM4 or TRPC3 channels, likely mediating/activating ICAN, plays a role in respiratory pattern formation but not in respiratory rhythm generation per se. The present study, that combines several complementary approaches, extends the current knowledge on the role of ICAN towards respiratory pattern formation and its contribution in controlling motoneuronal activity. Nevertheless the data as they are presented raise some concerns that are detailed below.

Major comments:

- Abstract: It is not clearly stated in the abstract what is the potential role of TRPC3 and why it is also examined in the study. Also the word “putative” is used three times which renders the overall

- Immuno data presented in Figure 2 show that the proteins of both channels are expressed by glutamatergic and glycinergic preBötC neurons. How was the preBötC region anatomically defined for cell counting? This should be specified in the M&M section.

More importantly, because both glutamatergic and glycinergic respiratory neurons express TRPM4/TRPC3, they are all affected by the pharmacological treatments applied to target ICAN. It would be interesting if the authors could include in the discussion their interpretation of how affecting these two cell classes could result in the overall effect observed. In other words what is the respective contribution of blocking excitatory versus inhibitory inspiratory neurons in the process?

- Immunostaining and Fig 1: Wouldn't it be possible to use a different primary antibody for either TRPM4 or TRPC3 in order to be able to perform a triple staining allowing direct testing of the possibility for the two channels to be expressed in the same neuron, as later shown with the single-cell RT-PCR protocol for mRNAs?

- Whole-cell patch clamp recordings: at several places in the text and also in Material and Methods section authors described that they have performed such recordings but not a single trace is shown in the entire paper. For example it would be extremely useful to illustrate the two types of neurons investigated with single-cell RT-PCR. Indeed, especially for readers not expert in the field, it is particularly important to show what the discharge pattern of a respiratory glycinergic neuron is. This does not require additional experiments as neurons have been harvested after being functionally identified during the patch clamp recording session (traces are therefore already recorded). In addition, and in a more general manner, it would have been useful to show what is the effect of the drugs on the discharge pattern of hypoglossal motoneurons and preBötC interneurons (either glutamatergic or glycinergic). If such recordings are available providing illustrations would strengthen the paper as these data could then be correlated to XII nerve and preBötC network recordings.

-Single-cell RT-PCR: The authors claimed that all harvested neurons were initially identified as inspiratory ones as they exhibited rhythmic fluorescent changes in phase with the XII nerve activity. VgluT2 and GlyT2 positive neurons were unambiguously considered as glutamatergic and glycinergic neurons, respectively. However I have problems with neurons expressing GAD67 only. I wonder whether this is correct to consider such cells as glycinergic?

- Figures 7 and 9 and associated results: The authors described a decrease in fluorescence over time during pharmacological treatment. How can they be sure that this is not due to photobleaching? For instance did they perform control experiments in which calcium imaging is done over a comparable period but without any drug application? Another experiment that should be done is performing calcium imaging at the level of the XII nucleus simultaneously with the electrophysiological recording of XII nerve activity. Then if a decrease in amplitude of both electrical and fluorescent signals is observed it would be a good argument against photobleaching. A comparable series of experiments could also be done at the preBötC level: field recording at the surface of the slice while calcium imaging in the contralateral network.

- Not being familiar with the in situ preparation it is unclear to me how the preBötC activity was recorded (line 583)?

- Slices preparations were obtained from newborn rats and mice (first post-natal week). But it has been published that the number of ICAN-dependent pacemakers increases during the post-natal period, becoming more significant after P8 (Pena et al., 2004; Del Negro et al., 2005). Why did the authors choose this developmental stage that does not seem to be the more appropriate one? Didn't they perform experiments on slice preparations obtained at older stages? If yes did they observe similar results?

Minor comments:

- Please justify somewhere why experiments have been done on both rats and mice?

- page 17-18: The first paragraph of the result section does not seem useful to me.

- page 19, lines 420-422: it would be fair to also cite Morgado-Valle et al., 2010.

- Long-term recordings of XII nerve activity: did the authors performed control experiments in which the nerve activity is recorded for a long time without any pharmacological application to make sure that the effect observed in the study is not simply due to a leak in the suction?

- line 776: Chevalier et al., 2016 did not include newborn animals data if I am correct. Please remove the reference.

- line 795: not sure the sentence is correct.

- Figure 1: the ChAT label is missing in the left panels E and F. Also it is difficult to recognize the XII nucleus in the sample provided in F for the mouse. Would it be possible to use a slice that includes the fourth ventricule (V4) as done in E, thus allowing the unambiguous identification of immunolabeled neuronal population as that of the XII nucleus?

## Author Response

Authors' responses to the Editor and Reviewers' suggestions for revision

Synthesis Statement for Author:

This manuscript was seen by two reviewers, both of whom were positive about the results and the importance of the findings for the field. That said, each raised a number of concerns that are provided below. The majority of these can be addressed by revising the manuscript for further detail and better clarity. When preparing a revised manuscript, please provide examples of whole cell recordings from the pre-BÃ¶tzinger cell types used for PCR, preferably also illustrating effects of the different blockers on those cells (and on hypoglossal motoneurons). In terms of additional control experiments, some assurance that decreases in calcium fluorescence or nerve activity are due to drug-associated effects on the channels (rather than photobleaching or some time-dependent technical artifact) should be provided. Finally, please provide more information on antibody specificity; the simple reference to “no primary” controls is inadequate for reagent validation.

Authors' response:

We thank the Editor and the Reviewers for recognizing the importance of our study in the field. We also appreciate the positive evaluation and very helpful suggestions for revision. We have revised the manuscript accordingly to address all of the concerns as a point-by-point reply detailed below. Briefly, we now provide representative examples of whole-cell recordings from the preBÃ¶tC excitatory and inhibitory inspiratory neurons used for PCR in the revised Figure 3. We also provide the results of control experiments in the Results section (page 24, line 521 and page 26, line 568) including a new panel A in Figure 8. In addition, we completely agree that information on antibody specificity is a very important issue, and therefore we have added the following statements regarding antibody specificity in the Materials and Methods section (page 7, line 131): “We verified the specificity of these TRPM4 and TRPC3 antibodies by confirming the absence of immunoreactivity in the mouse medullary tissue sections with the primary antibody that was preincubated for 1 hour at room temperature with saturating concentrations (10:1) of the antigenic blocking peptide (TRPM4: ab65597, Abcam, TRPC3: ACC-016, Alomone Labs). We also note that the specificity of the same TRPM4 and TRPC3 antibodies as those we used has been confirmed in a TRPM4 knockout mouse (Schattling et al., 2012) and a TRPC3 knockout mouse (Feng et al., 2013), respectively”.

Reviewer #1:

Advances the Field:

This report provided some evidences that TRPM4 and TRPC3 could be involved in the inspiratory burst generation.

Comments to the Authors:

This report provided some evidences that TRPM4 and TRPC3 could be involved in the inspiratory burst generation. This study was well designed and documented. Although results are rather clear and interesting, further explanations would be needed in some parts and some discussion seems to be missed. My comments are below.

Authors' response:

We thank this Reviewer for recognizing that our study was well designed and documented. We also thank the Reviewer for the highly constructive suggestions, and we have revised the manuscript to address all of the concerns raised.

Introduction:

Reviewer:

Page 4, para 2- Page 5: Authors actually mentioned results here with some discussion. The description seems to be too much for the introduction and to include those that may be not very conclusive. Authors should rewrite these parts more concisely as suitable for introduction.

Authors' response:

We have revised this part of Introduction as indicated below, including removal of some results, discussion, and conclusive description as suggested by the reviewer.

“TRPM4 and TRPC3 channels have been identified by mRNA/protein expression in tissue harvested from the mouse pre-BÃ¶tC region (Crowder et al., 2007; Ben-Mabrouk and Tryba, 2010), although not demonstrated to be expressed in functionally identified respiratory neurons. Here we examined, by single-cell RT-PCR, expression of TRPM4 and TRPC3 mRNA in functionally identified glutamatergic, glycinergic, and GABAergic inspiratory pre-BÃ¶tC neurons and also inspiratory motoneurons. We also examined neuronal channel expression by antibody labeling in the pre-BÃ¶tC region and motor nuclei. We then investigated if these channels contribute to respiratory circuit activity by pharmacological experiments with the selective channel inhibitors 9-phenanthrol for TRPM4 (Guinamard et al., 2014) and 3-pyrazole for TRPC3 (Kiyonaka et al., 2009) in neonatal rat and mouse slice preparations with rhythmically active pre-BÃ¶tC and respiratory motor circuits in vitro. We also analyzed perturbations of pre-BÃ¶tC excitatory circuit activity by dynamic Ca2+ imaging of inspiratory glutamatergic pre-BÃ¶tC neurons with a genetically-encoded Ca2+ sensor (Chen et al., 2013) in transgenic mice. We show that amplitudes of inspiratory pre-BÃ¶tC neuronal activity, and the correlated amplitudes of motoneuronal output in vitro, are significantly reduced by TRPM4 and TRPC3 channel inhibitors. The pharmacological profile of inspiratory activity attenuation by inhibiting TRPM4 activation matched that with another proposed blocker of ICAN (flufenamic acid, FFA), consistent with the concept that TRPM4 mediates ICAN (Launay et al., 2002). In all cases, the attenuation of inspiratory circuit activity occurred without significant perturbations of the frequency of the inspiratory rhythm. ”

Methods:

Reviewer:

Page 11, para 2: Regarding extracellular population activity from the pre-BÃ¶tC in the perfused in situ preparation, was it performed by the dorsal approach? How did you determine the precise region of the pre-BÃ¶tC?

Authors' response:

Yes, we performed extracellular recording by a dorsal approach. We determined the precise location of the pre-BÃ¶tC as described below.

We first defined the anatomical coordinates for the pre-BÃ¶tC location by mapping neuronal activity of medullary respiratory neurons in the ventral respiratory column. The pre-BÃ¶tC is readily identified by a characteristic pattern of pre-inspiratory/inspiratory activity, and the BÃ¶tC has a characteristic profile of post-inspiratory and augmenting expiratory activities, whereas rVRG has a known profile of primarily augmenting inspiratory population activity. Therefore, the pre-BÃ¶tC region is distinguishable from the more rostral BÃ¶tC region, and also the more caudal rVRG region. Based on these predetermined coordinates, we positioned the recording microelectrode by a computer-driven 3-dimensional micromanipulator in the pre-BÃ¶tC region defined by its signature of pre-inspiratory/inspiratory activity.

We have added the following statement in Materials and Methods (page 12, line 248): “Extracellular population activity from the pre-BÃ¶tC in the perfused in situ preparations was also recorded by a dorsal approach with a fine-tipped glass pipette (3 - 5 MΩ resistance) filled with 0.5 M sodium acetate (Sigma), or in some cases with a tungsten microelectrode (3 - 4 MΩ), which was positioned by a computer-controlled 3-dimensional micromanipulator (MC2000, MÃ¤rzhÃ¤user). The precise location of the pre-BÃ¶tC was determined by the anatomical coordinates that we initially defined by mapping neuronal activity of medullary respiratory neurons within the ventral respiratory column. The pre-BÃ¶tC is readily identified by a characteristic pattern of preinspiratory/inspiratory population activity, and distinguishable from the more rostral BÃ¶tC region, which has a characteristic profile of post-inspiratory and augmenting expiratory activities, and from the more caudal rVRG region, which has an established profile of augmenting inspiratory population activity”.

Results:

Reviewer:

Page 25, para 1, the last part: Authors described non-significant changes in normalized fR after application of antagonists. However, data of Fig. 9 showed apparent decrease of fR. Was this example not typical?

Authors' response:

We understand the reviewer's critique that fR (inspiratory frequency) appears to be somewhat reduced after the 9-phenanthrol application. However, there were no apparent changes of fR in this experiment (98.8% of the control value at 20 min). However, we agree that the particular data segment in the original Figure 9B may potentially give the wrong impression and is not representative of the overall trend in this experiment. We have therefore revised this figure with a data segment that is visually consistent with the conclusive summary results shown in Fig. 8 that revealed no significant changes in fR over the entire time course of the experimental measurements.

Reviewer:

Page 26, para 3, “yet less than non-specific concentrations of 9-phenanthrol”: What was nonspecific concentration? What responses were induced if you use non-specific concentration?

Authors' response:

As reported in the literature (e.g., Simard et al., 2012), higher concentration (100 ÂµM) of 9phenanthrol inhibits L-type Ca2+ currents and the delayed outward rectifier K+ current as nonspecific effects, while lower concentrations (10 ÂµM) of 9-phenanthrol caused no effects on these currents in mouse ventricular cardiomyocytes. Although these results suggest that high concentrations of 9-phenanthrol could inhibit not only TRPM4 channel, but also other channels, we have no evidence that the same concentration could apply to brainstem respiratory neurons under our experimental conditions. What we meant here was that we carefully chose the concentration for the pharmacological studies to avoid the possible non-specific effects. But, we did not analyze in detail the effect of higher concentration of the drugs in our study. We have removed this statement to avoid any confusion. However, we also note that we observed “complete” cessation of inspiratory XII motor output in some cases with the highest concentration (50 ÂµM) of 9-phenanthrol that we used.

Discussion:

Reviewer:

Results from RT-PCR of identified neurons showed fairly small number of inhibitory pre-BÃ¶tC neurons were TRPM4 and/or TRPC3 positive, suggesting that many inhibitory neurons are TRPM4 and/or TRPC3 negative, i.e. directly insensitive to the channel antagonists (is it correct?). Do you suggest any functional role of these inhibitory inspiratory neurons? These inhibitory neurons may contribute to perturbation of burst activity during application of the channel blockers (by being directly not sensitive to the drugs).

Authors' response:

Our sample of inhibitory inspiratory pre-BÃ¶tC neurons for RT-PCR experiments was not sufficient to allow the conclusion that many inhibitory neurons are TRPM4 and/or TRPC3 negative. In addition, sampling of pre-BÃ¶tC inspiratory neurons for RT-PCR probe was not completely random in terms of neurotransmitter phenotypes. In our experiments employing midline injection of Ca2+sensitive dye, our harvest of the cytoplasm was limited to pre-BÃ¶tC inspiratory neurons that have contralateral axonal projections. These types of pre-BÃ¶tC inspiratory neurons have mainly (80%) a glutamatergic phenotype (Koizumi et al., 2013).

We suggest that inhibitory inspiratory pre-BÃ¶tC neurons potentially play an important role in respiratory pattern generation, especially in the more intact in situ preparations, although the contribution of these neurons with or without TRPM4 and TRPC3 channel expression to perturbations of pre-BÃ¶tC activity and respiratory frequency remain to be identified. According to our respiratory central pattern generation (CPG) network model (e.g., Rubin et al., 2009b), different types (neurotransmitter phenotypes, active phase, bursting pattern) of respiratory interneurons in the pre-BÃ¶tC and the BÃ¶tC are functionally interacting to generate a normal threephase pattern of respiratory neural activity. In this CPG network model, inhibitory inspiratory neurons in the pre-BÃ¶tC play an important role in respiratory pattern generation by interacting with inhibitory expiratory neurons in the BÃ¶tC. Detailed information on contributions of TRPM4 and TRPC3 channel activation in different types of respiratory (especially inhibitory) neurons remain to be clarified.

We have added the following statement in Discussion (page 36, line 808) to address this issue and also another issue below (Pyr3 induced a decrease in fR in rat in situ preparation): “According to the respiratory central pattern generation (CPG) network model based on experimental analyses with in situ preparations (Rubin et al., 2009b), different types (e.g., neurotransmitter phenotypes, active phase, bursting pattern) of respiratory interneurons in the pre-BÃ¶tC and BÃ¶tC are functionally interacting to generate a normal three-phase pattern of respiratory neural activity. Our experimental results of different effects of TRPM4 or TRPC3 channel inhibition on fR in the more intact in situ preparations (Fig. 12) suggest different sensitivity to TRPM4 or TRPC3 channel inhibitors among different types of respiratory neurons in the CPG circuits. Contributions of TRPM4 and TRPC3 channel activation in different types of excitatory and/or inhibitory respiratory neurons remain to be clarified.”

Reviewer:

Although authors discussed some difficulty of detailed mechanisms of contribution of TRPM4/ICAN or TRPC3 on the inspiratory burst generation, analyses of electrophysiological properties (spike frequency, drive potential, etc.) obtained by whole-cell recordings of inspiratory neurons might be helpful for understanding the inhibitory effects.

Authors' response:

We agree with the reviewer, but we have not yet performed the single-cell level analysis defining the detailed conductance mechanisms underlying contributions of TRPM4/ICAN or TRPC3 to inspiratory burst generation, which is a much larger project that we will pursue in the future.

Reviewer:

What is a main factor (e.g. number of active cells, burst amplitude of each active cells or others) for decrease of XII burst amplitude and field deltaF amplitude of calcium signal in the pre-BÃ¶tC by channel antagonists?

Authors' response:

We have concluded that the critical population of pre-BÃ¶tC excitatory neurons contributes to decrease of the XII burst amplitude as well as the field ÎF amplitude of calcium signals in the preBÃ¶tC. From our analyses on perturbations of Ca2+-transients of individual pre-BÃ¶tC glutamatergic neurons (cell ÎF) in relation to perturbations at the pre-BÃ¶tC population-level (field ÎF), some inspiratory neurons were sensitive to the inhibitors and the amplitude of their calcium transients was decreased or disappeared, while other inspiratory neurons were insensitive to the inhibitors and they showed no change in amplitude (Fig. 10). Furthermore, we established that the field ÎF reflects the mean cell ÎF of sets of individual excitatory inspiratory neurons (identity lines in Fig. 10). We also note that we found significantly correlated, linear relationships (Fig. 8) between the amplitude of the field ÎF in the pre-BÃ¶tC and the amplitude of XII inspiratory activity. These results suggest that the number of active pre-BÃ¶tC inspiratory cells as well as burst amplitude of each active pre-BÃ¶tC cell after application of the channel blockers are important factors for the overall reduction of pre-BÃ¶tC field amplitude and decrease of XII burst amplitude, as the reviewer mentioned.

We have added the following statement in Discussion (page 34, line 768): “Our results suggest that the number of active pre-BÃ¶tC inspiratory cells as well as burst amplitude of each active preBÃ¶tC neuron after application of the channel inhibitors are important factors contributing to the overall reduction of pre-BÃ¶tC field ÎF and decrease of XII burst amplitude.”

Reviewer:

Pyr3 induced a decrease in fR in rat in situ preparation. Please discuss about this result.

Authors' response:

We thank the reviewer for suggesting the additional discussion about our interesting experimental results. Pyr3 (TRPC3 channel inhibitor) induced a decrease in fR in rat in situ preparation, although Pyr3 had no significant effects on fR in mouse in situ preparation, while TRPM4 channel inhibition produced the same results of increase in fR in both rat and mouse in situ preparations. These results potentially suggest some species-related difference between rats and mice of the TRPC3 channel contribution to respiratory pattern generation. As discussed above, according to our respiratory central pattern generation (CPG) network model (e.g., Rubin et al., 2009b), different types of respiratory neurons in the pre-BÃ¶tC and the BÃ¶tC are functionally interacting to generate a normal three-phase pattern of respiratory neural activity. In these CPG networks, attenuating the activity of inhibitory post-I neurons in the BÃ¶tC can cause an increase of inspiratory frequency. Therefore, one possible explanation for the above in situ experimental results would be that (1) the TRPM4 channel inhibitor also potently reduces the amplitude of BÃ¶tC inhibitory post-I neuron population activity in both rats and mice, and therefore causes an increases the fR, and (2) the TRPC3 inhibitor also affects activity of other neuron types leading to a more complex pattern of circuit interactions and modulation that appears to differ from the TRPM4 inhibitor regarding fR perturbations in mice and especially rats. We need to analyze the drug effects on different types of respiratory neurons with cellular recordings in the in situ preparations in a future study. Regardless, the results are clear that blocking these channels does not disrupt rhythm generation.

As noted above, we have added a statement in Discussion (page 36, line 808) about the possibility of different sensitivity to TRPM4 and TRPC3 channel inhibitors among different types of respiratory neurons in the CPG circuits and the need to further clarify contributions of TRPM4 and TRPC3 channel activation in different types of respiratory neurons.

Figures:

Reviewer:

Fig. 1 and 2: Although I believe that these regions are in the pre-BÃ¶tC, some atlas (with lower magnification?) indicating landmarks of the regions would be helpful for better understanding of general readers who are not familiar in this field.

Authors' response:

As suggested by the reviewer, we have added a lower magnification panel (Fig. 1A) to show the landmarks of the regions. We also revised the legend for this figure accordingly.

Reviewer:

Fig. 2: Why is tdTomato expressed by 'green'? ('Red' is more natural). Please add some explanations or show also gray scale figures (?).

Authors' response:

We have changed the colors of panels in Fig. 2 as recommended.

Reviewer:

Fig. 3: More delated explanations for this figure are needed; column labels, maker labels, etc. I understood that the cytoplasm was treated separately for RT-PCR to identify five types of molecules as indicated in each column. Is it OK?

There is no example of the whole-cell recording from inspiratory neurons in any figures. Indication of some examples somewhere (e.g. in Fig. 3) with recording sites might be better.

Authors' response:

We have added more detailed information on the Figure 3. The final PCR amplification reaction was done separately for each gene of interest- i.e., as we originally indicated in Materials and Methods (page 14, line 297): “The second round of PCR amplification was performed as individual reactions with primers for TRPM4, TRPC3, VgluT2, glyT2, or GAD67.”

We have also added examples of whole-cell recordings from representative excitatory and inhibitory inspiratory neurons and additional information regarding methods in the revised Fig. 3 and legend.

Reviewer #2:

Comments to the Authors:

The present study investigates the potential role of TRPM4, a putative molecular substrate for the calcium-activated non-selective cation current ICAN, and TRPC3, involved in regulating calciumrelated signaling in neurons, in contributing in respiratory pattern and/or rhythmogenesis. Using a combination of electrophysiological neuronal activities recordings, calcium imaging, immunostainings and single-cell multiplex RT-PCR performed on different preparations (in vitro brainstem slice preparations, arterially perfused in situ brainstem-spinal cord preparations) and different animal models (rats or mice) the authors showed that pharmacological blockade of ICAN by different agents targeting either TRPM4 or TRPC3 channels or the ICAN current itself, leads to a reduction of the amplitude of inspiratory interneuronal (preBÃ¶tC neurons) and motoneuronal (hypoglossal motoneurons) activity without significantly affecting the frequency of the fictive inspiratory rhythm. The authors conclude that activation of TRPM4 or TRPC3 channels, likely mediating/activating ICAN, plays a role in respiratory pattern formation but not in respiratory rhythm generation per se. The present study, that combines several complementary approaches, extends the current knowledge on the role of ICAN towards respiratory pattern formation and its contribution in controlling motoneuronal activity. Nevertheless, the data as they are presented raise some concerns that are detailed below.

Authors' response:

We thank this Reviewer for recognizing that our study extends the current knowledge on the role of ICAN. We have revised the manuscript to address all of the reviewer's major and minor concerns.

Major comments:

Reviewer:

- Abstract: It is not clearly stated in the abstract what is the potential role of TRPC3 and why it is also examined in the study. Also, the word “putative” is used three times which renders the overall.

Authors' response:

We have revised the abstract as the reviewer suggested by adding the following statement with regard to potential involvement and roles of TRPC3 channel in regulating the pre-BÃ¶tC inspiratory neurons: “Another TRP channel, TRPC3, which mediates Na+/Ca2+ fluxes, may be involved in regulating Ca2+-related signaling including affecting TRPM4/ICAN in respiratory pre-BÃ¶tC neurons.” We have also deleted the word “putative” on two occasions from the abstract.

Reviewer:

- Immuno data presented in Figure 2 show that the proteins of both channels are expressed by glutamatergic and glycinergic preBÃ¶tC neurons. How was the preBÃ¶tC region anatomically defined for cell counting? This should be specified in the M&M section.

Authors' response:

We have added the following statement in Materials and Methods section (page 7, line 145): “For tallying the number of TRPM4 and TRPC3 channel antibody-labeled neurons as presented in Results, we counted labeled neurons within a region (300 - 400 Âµm diameter depending on animal size) encompassing the pre-BÃ¶tC, BÃ¶tC, or rVRG in the coronal plane on each side of the medulla. Multiple coronal sections (4 - 6) from different levels along the rostro-caudal extent of these regions were used for the analysis to produce the total regional tally of labeled neurons”.

Reviewer:

More importantly, because both glutamatergic and glycinergic respiratory neurons express TRPM4/TRPC3, they are all affected by the pharmacological treatments applied to target ICAN. It would be interesting if the authors could include in the discussion their interpretation of how affecting these two cell classes could result in the overall effect observed. In other words what is the respective contribution of blocking excitatory versus inhibitory inspiratory neurons in the process?

Authors' response:

We thank the reviewer for raising this important point. Our interpretation for the respective contribution of TRPM4/ICAN or TRPC3 channel inhibition in excitatory and inhibitory inspiratory neurons is that (1) our dynamic Ca2+ imaging experiments (Figs. 7 - 8) on excitatory (VgluT2positive) pre-BÃ¶tC inspiratory neurons suggest that inhibition of TRPM4/ICAN or TRPC3 in excitatory pre-BÃ¶tC inspiratory neurons primarily contributes to the amplitude decrease of inspiratory motor outputs in vitro, but not to inspiratory rhythm generation, and (2) our in situ experiments (Fig. 12) suggest that blocking TRPM4 or TRPC3 channels in inhibitory BÃ¶tC expiratory neurons may have caused perturbations of post-I activity in vagus nerves and respiratory frequency. In summary, we suggest TRPM4/ICAN or TRPC3 activation plays an important role in regulating activity of excitatory and inhibitory respiratory neurons particularly in the intact in situ CPG circuits for respiratory pattern generation.

We have added the following statement in Discussion (page 37, line 817): “Our results suggest that inhibiting TRPM4/ICAN or TRPC3 in excitatory pre-BÃ¶tC inspiratory neurons primarily contributes to the amplitude decrease of inspiratory motor outputs in vitro and in situ, while inhibiting TRPM4 or TRPC3 channels in inhibitory neurons, possibly BÃ¶tC expiratory neurons, in the more intact in situ circuits causes perturbations of post-I activity (e.g., Marchenko et al. 2016) and therefore respiratory frequency. In summary, we suggest that endogenous activation of TRPM4/ICAN or TRPC3 plays an important role in regulating activity of excitatory and inhibitory respiratory neurons, the latter particularly in the intact in situ CPG circuits for inspiratoryexpiratory pattern generation.”

Reviewer:

- Immunostaining and Fig 1: Wouldn't it be possible to use a different primary antibody for either TRPM4 or TRPC3 in order to be able to perform a triple staining allowing direct testing of the possibility for the two channels to be expressed in the same neuron, as later shown with the singlecell RT-PCR protocol for mRNAs?

Authors' response:

We thank the reviewer for the useful suggestion of triple staining, but as previously stated in Materials and Methods (page 7, line 130), the TRPM4 and TRPC3 channel antibodies used were both produced in rabbits. We tested a number of antibodies for TRPM4 and TRPC3 channels and tried triple staining, but at the time we found only the above antibodies worked well consistently for the pre-BÃ¶tC neurons. The results from our immunohistochemical experiments clearly show that many of the pre-BÃ¶tC neurons express TRPM4 and TRPC3 channel antibody, although it does not show the information of the two channels expressed in the same neuron, which was revealed by the single-cell RT-PCR experiments.

Reviewer:

- Whole-cell patch clamp recordings: at several places in the text and also in Material and Methods section authors described that they have performed such recordings but not a single trace is shown in the entire paper. For example, it would be extremely useful to illustrate the two types of neurons investigated with single-cell RT-PCR. Indeed, especially for readers not expert in the field, it is particularly important to show what the discharge pattern of a respiratory glycinergic neuron is. This does not require additional experiments as neurons have been harvested after being functionally identified during the patch clamp recording session (traces are therefore already recorded). In addition, and in a more general manner, it would have been useful to show what is the effect of the drugs on the discharge pattern of hypoglossal motoneurons and preBÃ¶tC interneurons (either glutamatergic or glycinergic). If such recordings are available providing illustrations would strengthen the paper as these data could then be correlated to XII nerve and preBÃ¶tC network recordings.

Authors' response:

We thank the reviewer for this very useful suggestion. We have added representative examples of whole-cell recordings from the two types of neurons investigated with single-cell RT-PCR in the revised Fig. 3C. We currently do not have the experimental results of drug effects on electrophysiologically recorded discharge pattern of individual XII motoneurons or preBÃ¶tC interneurons. In this study, we were particularly interested in population-level activity and our calcium imaging data allowed the necessary correlations between single-cell and population activity at the level of the critical excitatory population in the pre-BÃ¶tC, and also the correlations between pre-BÃ¶tC excitatory population activity and XII motoneuronal activity.

Reviewer:

-Single-cell RT-PCR: The authors claimed that all harvested neurons were initially identified as inspiratory ones as they exhibited rhythmic fluorescent changes in phase with the XII nerve activity. VgluT2 and GlyT2 positive neurons were unambiguously considered as glutamatergic and glycinergic neurons, respectively. However, I have problems with neurons expressing GAD67 only. I wonder whether this is correct to consider such cells as glycinergic?

Authors' response:

The neurons expressing only GAD67 mRNA are GABAergic inhibitory neurons and are not considered to be glycinergic neurons. From previous studies (e.g., Kuwana et al., 2006; Koizumi et al., 2013), it is known that there are GABAergic inspiratory neurons in the pre-BÃ¶tC, although as shown in the latter study, there is also a substantial subpopulation of inhibitory neurons that coexpress GABA and glycine.

Reviewer:

- Figures 7 and 9 and associated results: The authors described a decrease in fluorescence over time during pharmacological treatment. How can they be sure that this is not due to photobleaching? For instance, did they perform control experiments in which calcium imaging is done over a comparable period but without any drug application? Another experiment that should be done is performing calcium imaging at the level of the XII nucleus simultaneously with the electrophysiological recording of XII nerve activity. Then if a decrease in amplitude of both electrical and fluorescent signals is observed it would be a good argument against photobleaching. A comparable series of experiments could also be done at the preBÃ¶tC level: field recording at the surface of the slice while calcium imaging in the contralateral network.

Authors' response:

We understand the reviewer's concern that a decrease in fluorescence over time during drug application could be due to photobleaching. However, we performed control experiments in which calcium imaging was done over the same period without any drug application. The results showed that there were no significant changes in pre-BÃ¶tC fluorescence activity with exactly the same protocol of image acquisition as the pharmacological experiments. We now show the results of these control experiments on ÎF, and also on XII amplitude and respiratory frequency in the revised Figure 8A. We have also added the following statement in Results section (page 26, line 568): “We performed control experiments (Fig. 8A, n = 5 mice) to test for possible photobleaching or time-dependent changes in population activity, in which calcium imaging was performed without any drug application with exactly the same protocol of image acquisition as the pharmacological experiments. The results showed that there were no significant changes in the pre-BÃ¶tC field ÎF (102 {plus minus} 3% of control at 20 min, Spearman correlation coefficient rs = 0.11; p = 0.62), integrated XII amplitude (99 {plus minus} 3%, rs = -0.078; p = 0.74), and respiratory frequency (100 {plus minus} 2%, rs = 0.092; p = 0.70)”.

We believe that these control experiment results give enough assurance that decreases of amplitude in calcium fluorescence of the pre-BÃ¶tC or of the XII nerve activity are due to drug-associated effects on the TRP channels rather than photobleaching or other technical artifacts. Therefore, we did not perform the calcium imaging at the level of the XII nucleus or field recording of pre-BÃ¶tC activity at the surface of the slice with calcium imaging in the contralateral network.

Reviewer:

- Not being familiar with the in situ preparation it is unclear to me how the preBÃ¶tC activity was recorded (line 583)?

Authors' response:

We have added the following statement in Materials and Methods (page 12, line 248): “Extracellular population activity from the pre-BÃ¶tC in the perfused in situ preparations was also recorded by a dorsal approach with a fine-tipped glass pipette (3 - 5 MΩ resistance) filled with 0.5 M sodium acetate (Sigma), or in some cases with a tungsten microelectrode (3 - 4 MΩ), which was positioned by a computer-controlled 3-dimensional micromanipulator (MC2000, MÃ¤rzhÃ¤user). The precise location of the pre-BÃ¶tC was determined by the anatomical coordinates that we initially defined by mapping neuronal activity of medullary respiratory neurons within the ventral respiratory column. The pre-BÃ¶tC is readily identified by a characteristic pattern of preinspiratory/inspiratory population activity, and distinguishable from the more rostral BÃ¶tC region, which has a characteristic profile of post-inspiratory and augmenting expiratory activities, and from the more caudal rVRG region, which has an established profile of augmenting inspiratory population activity.”

Reviewer:

- Slices preparations were obtained from newborn rats and mice (first post-natal week). But it has been published that the number of ICAN-dependent pacemakers increases during the post-natal period, becoming more significant after P8 (Pena et al., 2004; Del Negro et al., 2005). Why did the authors choose this developmental stage that does not seem to be the more appropriate one? Didn't they perform experiments on slice preparations obtained at older stages? If yes did they observe similar results?

Authors' response:

We appreciate the reviewer's critique that we need the analyses on older stages. To investigate the TRP channel/ICAN-dependent mechanism at an older developmental stage, we performed experiments from mature rats and mice by using the in situ brainstem-spinal cord preparations, which is even better, we believe, than the slice preparations in vitro at the older ages. We obtained similar results of a non-critical role of a TRPM4 channel/ICAN-dependent mechanism in rhythm generation, but a dominant role in pattern formation. We actually stated in the original Discussion (page 35, line 788): “Moreover, extending our analysis to mature animals was necessary since ICAN-dependent neuronal bursting, and accordingly potential involvement of TRPM4, has been proposed to contribute to inspiratory rhythm generation predominantly in mice older than P5 (Pena et al., 2004; Del Negro et al., 2005), although ICAN is postulated to contribute to formation of drive potentials generating inspiratory bursts throughout development (Del Negro et al., 2005).”

Minor comments:

Reviewer:

- Please justify somewhere why experiments have been done on both rats and mice?

Authors' response:

We agree that we should discuss this point. In the field, there are numerous studies on respiratory rhythm and pattern generation using rats (e.g. Gray et al., 2001; Onimaru and Homma, 2003; Koizumi and Smith, 2008) or mice (e.g. Thoby-Brisson and Ramirez, 2001; Pena et al., 2004; Del Negro et al., 2005). It is therefore necessary to compare these two species and establish commonality of Ca2+-based mechanisms for respiratory rhythm and pattern generation. Here, we demonstrated similar results from both rats and mice regarding TRP channel contributions to respiratory rhythm and pattern generation.

We have now provided a rationale in Introduction (page 4, line 76): “Comparative analyses for these two species was important because there are numerous studies on respiratory rhythm and pattern generation using rats (e.g., Gray et al., 2001; Onimaru and Homma, 2003; Koizumi and Smith, 2008) or mice (e.g., Thoby-Brisson and Ramirez, 2001; Pena et al., 2004; Del Negro et al., 2005), so it is necessary to establish commonality of Ca2+-based mechanisms for respiratory rhythm and pattern generation.”

Reviewer:

- page 17-18: The first paragraph of the result section does not seem useful to me.

Authors' response:

We have removed this paragraph, which is redundant with other parts of the Results section.

Reviewer:

- page 19, lines 420-422: it would be fair to also cite Morgado-Valle et al., 2010.

Authors' response:

We have added this reference (page 19, line 423) as recommended.

Reviewer:

- Long-term recordings of XII nerve activity: did the authors performed control experiments in which the nerve activity is recorded for a long time without any pharmacological application to make sure that the effect observed in the study is not simply due to a leak in the suction?

Authors' response:

Yes, we performed control experiments (n = 6 each in rats and mice), in which the XII nerve activity was recorded for 60 min without application of any pharmacological agents, and found no changes in amplitude of XII nerve activity after 60 min of recordings. Also, after all of the pharmacological experiments, we confirmed there were no leaks in the suction electrodes of the XII nerves. This XII nerve activity issue was also handled when addressing the photobleaching issue described above. We have added panel A in Fig. 8 about the results of control experiments for XII burst amplitude (XII Amp). We have also added the following statement in Results section (page 24, line 521): “We also performed control experiments (n = 6 each in rats and mice), in which the XII nerve activity was recorded for 60 min without application of any pharmacological agents, and found no significant changes in inspiratory burst amplitude of XII nerve activity (100 {plus minus} 3% in rats and 99 {plus minus} 4% in mice to the control value at 60 min; p = 0.36 in rats and p = 0.36 in mice, respectively).”

Reviewer:

- line 776: Chevalier et al., 2016 did not include newborn animals data if I am correct. Please remove the reference.

Authors' response:

We have removed this reference, which studied mouse embryonic respiratory networks.

Reviewer:

- line 795: not sure the sentence is correct.

Authors' response:

We have replaced the original statement by the following describing differences in fR perturbations between the TRPM4 and TRPC3 channel inhibitors in situ.

In Discussion (page 36, line 808): “According to the respiratory central pattern generation (CPG) network model based on experimental analyses with in situ preparations (Rubin et al., 2009b), different types (e.g., neurotransmitter phenotypes, active phase, bursting pattern) of respiratory interneurons in the pre-BÃ¶tC and BÃ¶tC are functionally interacting to generate a normal threephase pattern of respiratory neural activity. Our experimental results of different effects of TRPM4 or TRPC3 channel inhibition on fR in the more intact in situ preparations (Fig. 12) suggest different sensitivity to TRPM4 or TRPC3 channel inhibitors among different types of respiratory neurons in the CPG circuits. Contributions of TRPM4 and TRPC3 channel activation in different types of excitatory and/or inhibitory respiratory neurons remain to be clarified.”

Reviewer:

- Figure 1: the ChAT label is missing in the left panels E and F. Also, it is difficult to recognize the XII nucleus in the sample provided in F for the mouse. Would it be possible to use a slice that includes the fourth ventricle (V4) as done in E, thus allowing the unambiguous identification of immunolabeled neuronal population as that of the XII nucleus?

Authors' response:

We have added the ChAT labels in the left panels of E and F, and also replaced F with new panels that include the fourth ventricle (V4) as recommended.
